# Di-valent siRNA-mediated silencing of MSH3 blocks somatic repeat expansion in mouse models of Huntington’s disease

**DOI:** 10.1016/j.ymthe.2023.09.016

**Published:** 2023-09-25

**Authors:** Daniel O’Reilly, Jillian Belgrad, Chantal Ferguson, Ashley Summers, Ellen Sapp, Cassandra McHugh, Ella Mathews, Adel Boudi, Julianna Buchwald, Socheata Ly, Dimas Moreno, Raymond Furgal, Eric Luu, Zachary Kennedy, Vignesh Hariharan, Kathryn Monopoli, X. William Yang, Jeffery Carroll, Marian DiFiglia, Neil Aronin, Anastasia Khvorova

## Main text

(Molecular Therapy *31*, 1661–1674; June 2023)

In the originally published version of this article, the data for MSH2, PMS1, and PMS2 in Figure 6 were unintentionally duplicated. After reviewing the original data, the authors found that the MSH2 data had been accidentally copied and pasted into the PMS1 and PMS2 data cells during data analysis. The corrected figure has been provided below. The authors state that the conclusions of Figure 6 and the manuscript have not changed with this correction.

This article has been corrected online, and the authors apologize for any confusion this may have caused.

**Figure:**
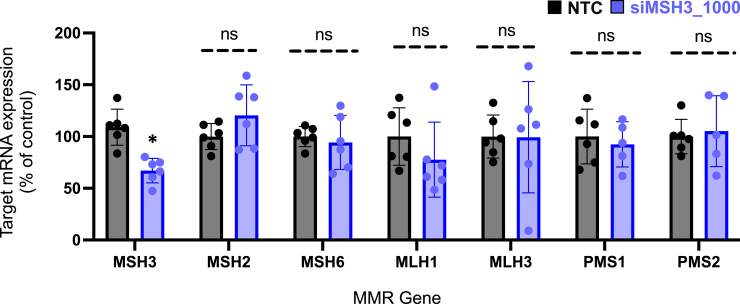
Figure 6. MSH3 silencing does not alter expression of additional mismatch repair genes (corrected)

**Figure:**
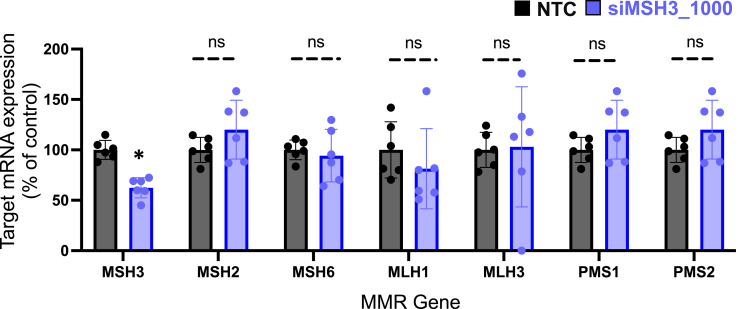
Figure 6. MSH3 silencing does not alter expression of additional mismatch repair genes (original)

