# Supplementary material for: Di-valent siRNA-mediated silencing of MSH3 blocks somatic repeat expansion in mouse models of Huntington’s disease
Source: Mol Ther. 2023 May 12;31(6):1661–74. doi: 10.1016/j.ymthe.2023.05.006 (PMC10277892; doi:10.1016/j.ymthe.2023.05.006)
Supplement: Document S1. Figures S1–S7 and Table S1 [file mmc1.pdf]

YMTHE, Volume *31*

Supplemental Information

Di-valent siRNA-mediated silencing of MSH3 blocks somatic repeat expansion in mouse models of Huntington's disease

Daniel O'Reilly, Jillian Belgrad, Chantal Ferguson, Ashley Summers, Ellen Sapp, Cassandra McHugh, Ella Mathews, Adel Boudi, Julianna Buchwald, Socheata Ly, Dimas Moreno, Raymond Furgal, Eric Luu, Zachary Kennedy, Vignesh Hariharan, Kathryn Monopoli, X. William Yang, Jeffery Carroll, Marian DiFiglia, Neil Aronin, and Anastasia Khvorova

SUPPLEMENTAL MATERIALS

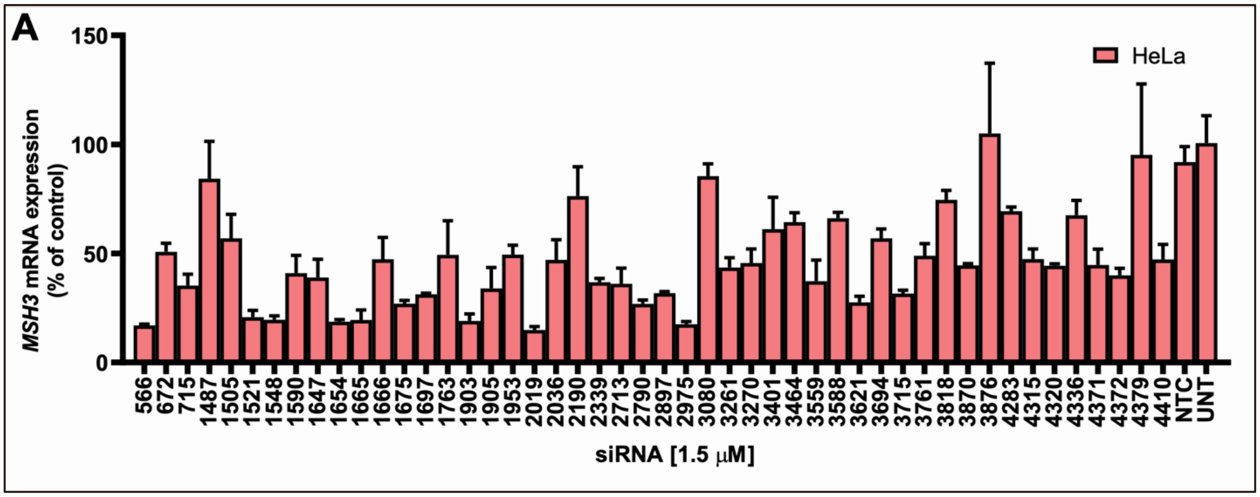

Figure S1: Identification of potent siRNA targeting *Msh3*. *In vitro* screen results for various MSH3-targeting sequences, aligned by 5'-3' location across the target gene. HeLa cells treated 1.5 μM siRNA for 72 hours. mRNA quantified with Quantigene assay and *Msh3* expression represented as abundance normalized to HPRT and relative to NTC.

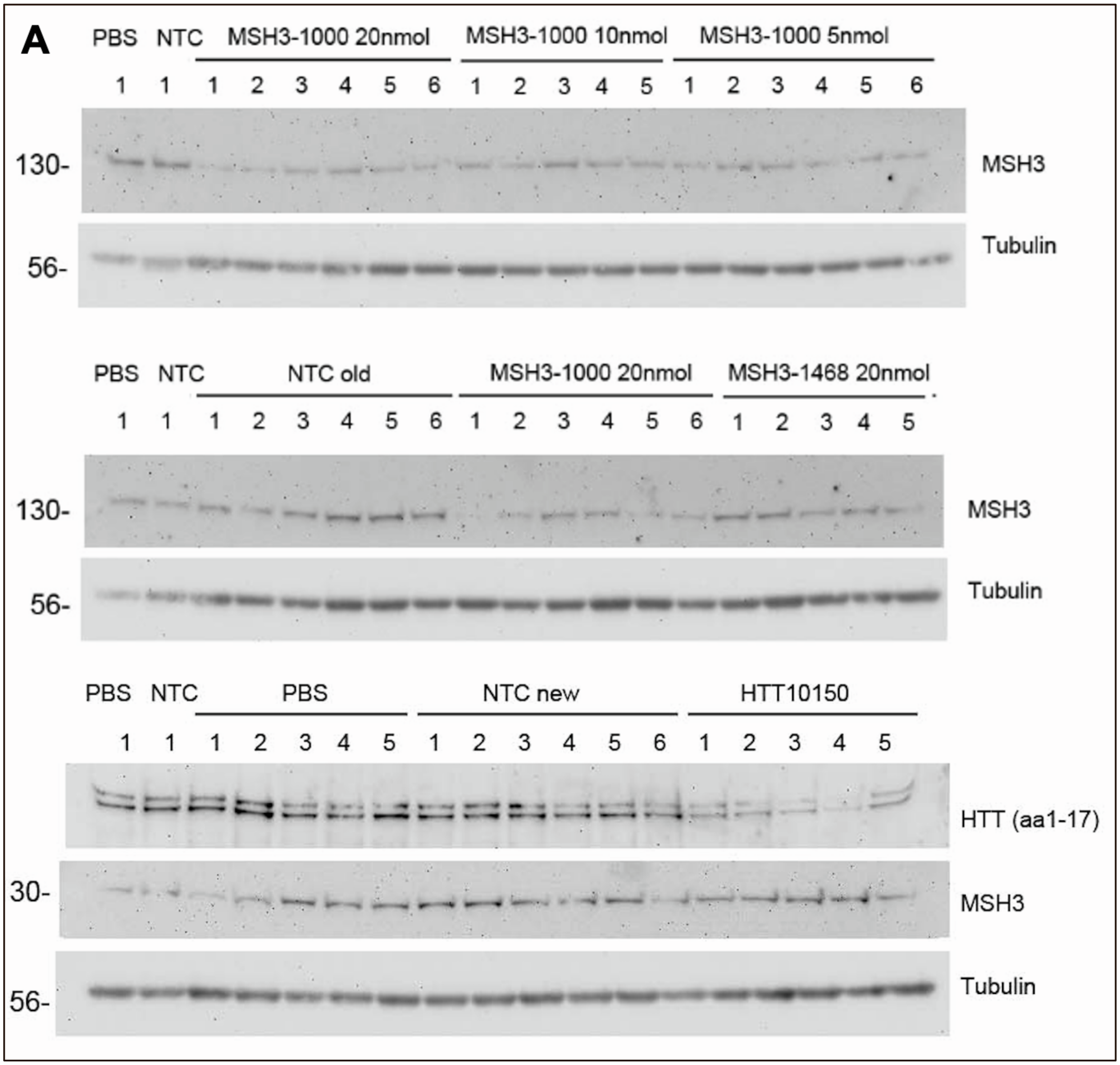

Figure S2: MSH3_1000 silences MSH3 *in vivo* two months post ICV injection in Hdh^{Q111} HD mice. Western blots used to quantify MSH3 and HTT protein expression in treated mice. HTT and MSH3 expression normalized to tublin loading control. Treatment group listed above each section. Molecular weight of target band reported on left. Western blot conditions reported in methods.

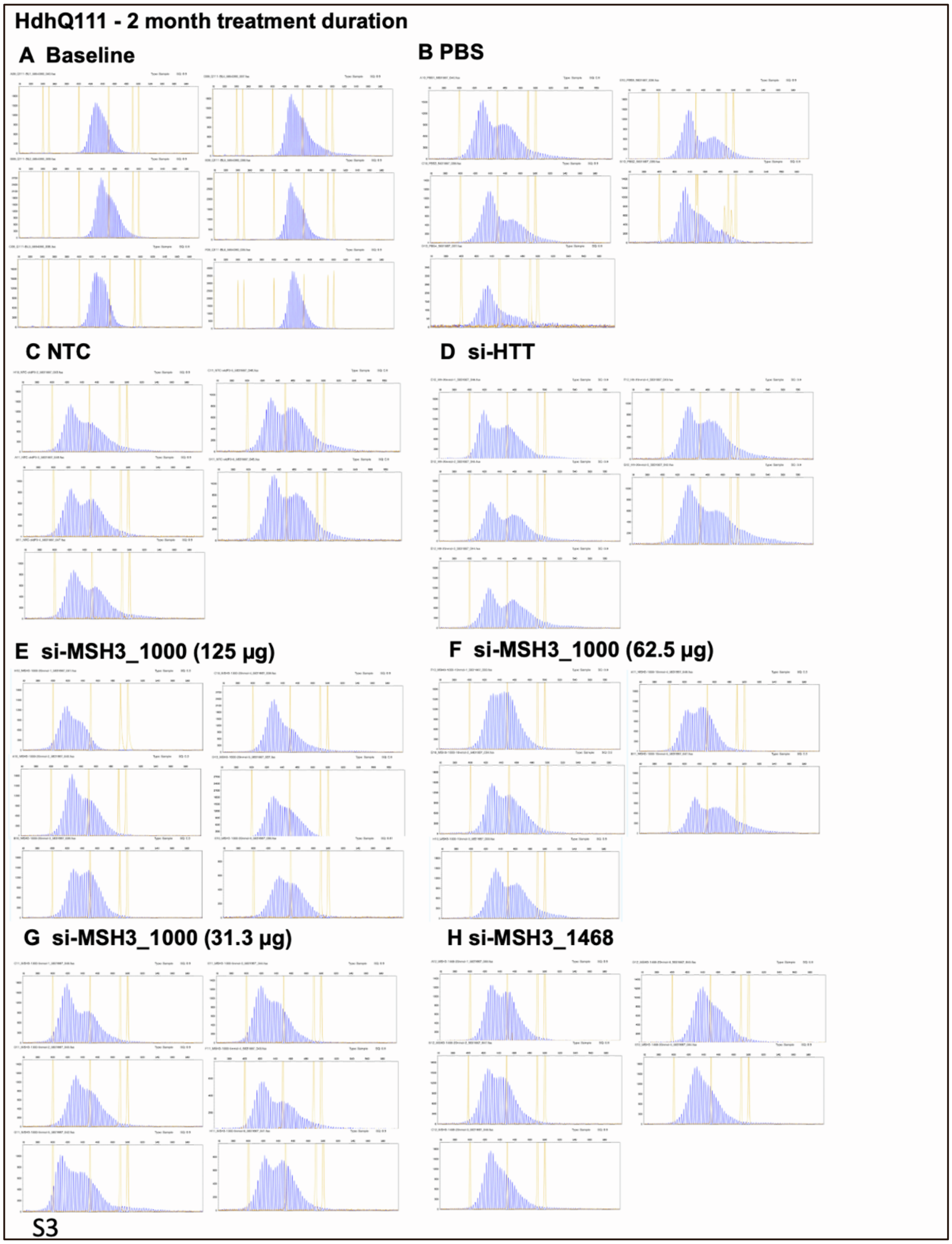

Figure S3: Fragment analysis traces indicate that siMSH3 blocks somatic repeat expansion in Hdh^{Q111} following two-month treatment. Fragment analysis traces used to quantify somatic repeat expansion instability index for (A) Baseline, (B) PBS, (C) NTC, (D) si-Htt (E) siMSH3_1000 (125 µg) (F) siMSH3_1000 (62.5 µg) (G) siMSH3_1000 (31.3 µg) (H) ssiMSH3_1468 (125 µg). X-asis of each chart is fragment length (base pairs). Y-axis is relative fluorescence units normalized to region of interest height. Orange lines are fragment ladders.

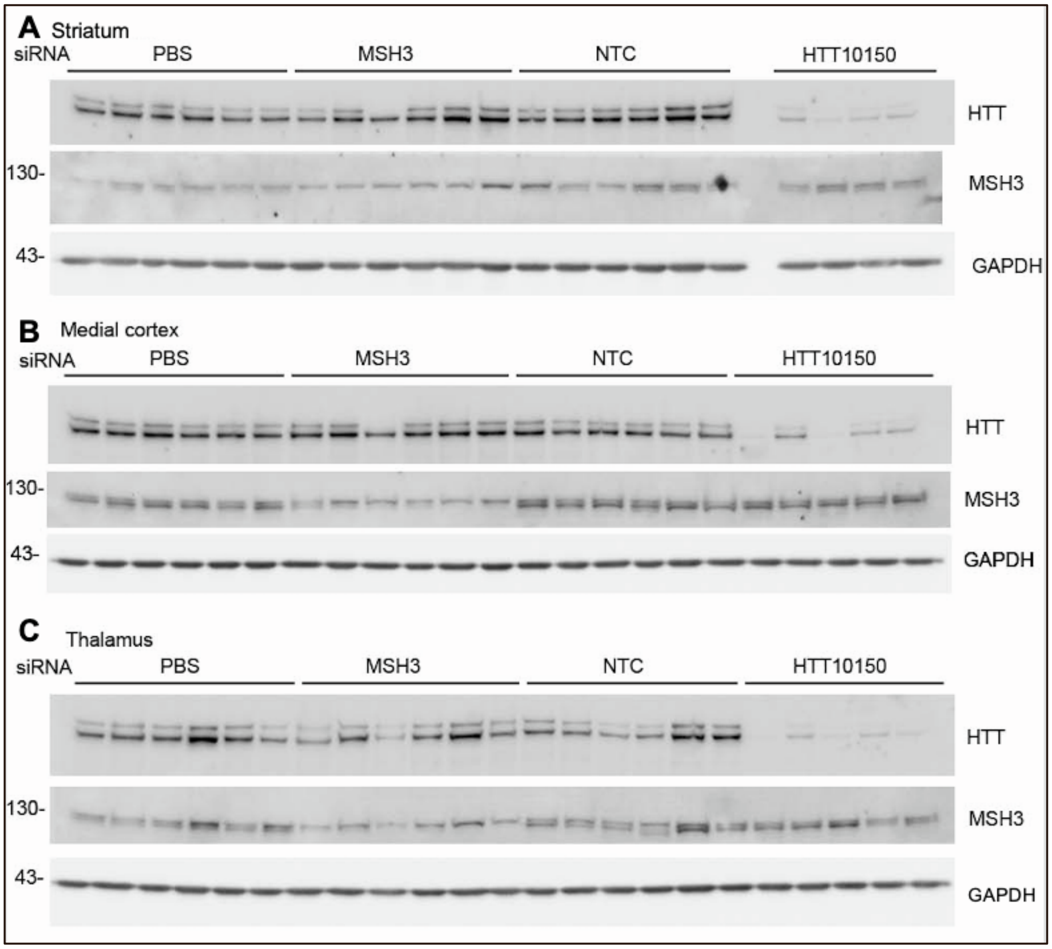

Figure S4: MSH3_1000 silences MSH3 *in vivo* two months post ICV injection in BAC-CAG HD mice. Western blots used to quantify MSH3 and HTT protein expression in **(A)** striatum, **(B)** medial cortex, **(C)** and thalamus. HTT and MSH3 expression normalized to GAPDH loading control. Treatment group listed above each section. Molecular weight of target band reported on left. Western blot conditions reported in methods.

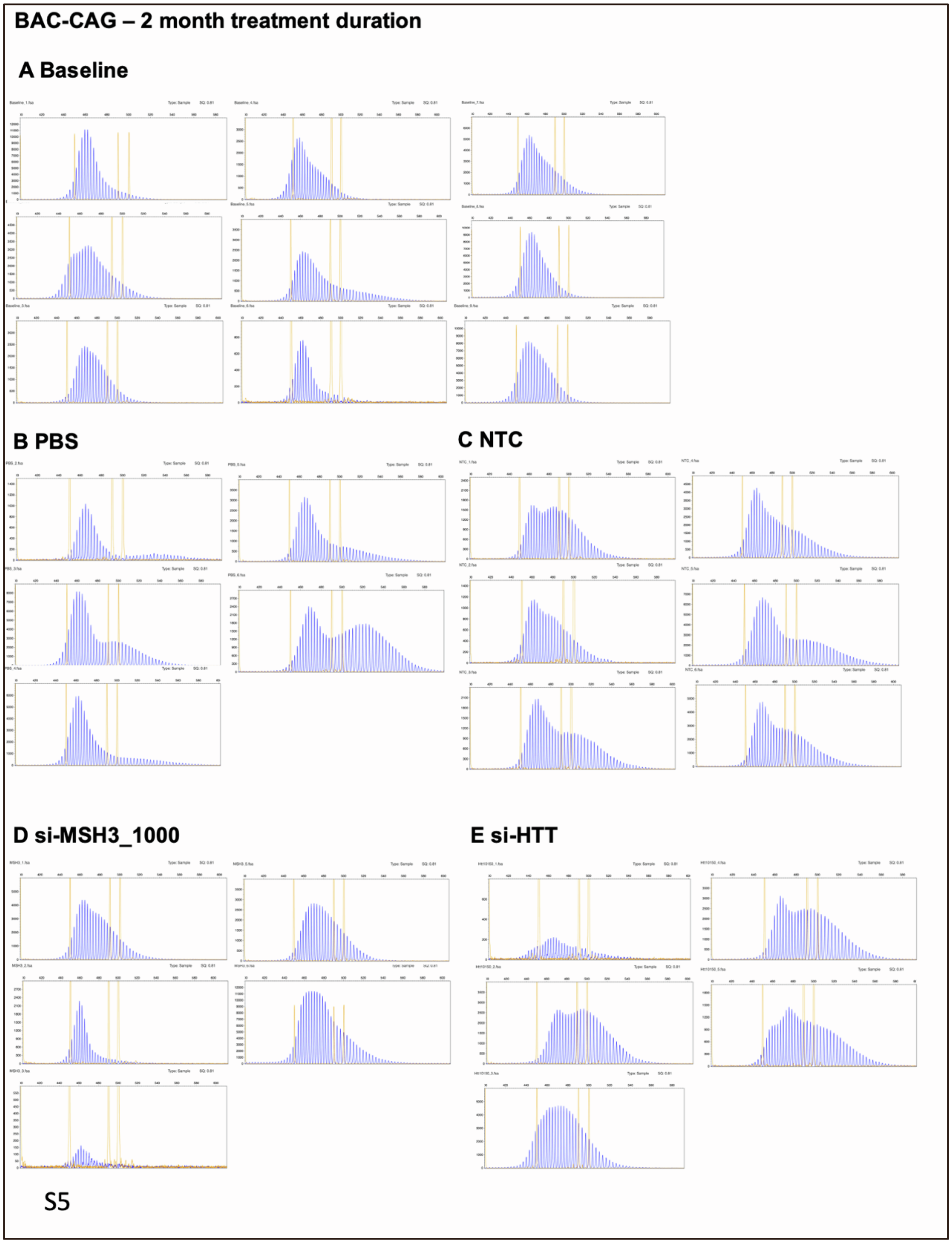

Figure S5: Fragment analysis traces indicate that siMSH3 blocks somatic repeat expansion in BAC-CAG following two-month treatment. Fragment analysis traces used to quantify somatic repeat expansion instability index for (A) Baseline, (B) PBS, (C) NTC, (D) siMSH3_1000 (E) si-Htt. X-axis of each chart is fragment length (base pairs). Y-axis is the relative fluorescence units normalized to region of interest height. Orange lines are fragment ladders.

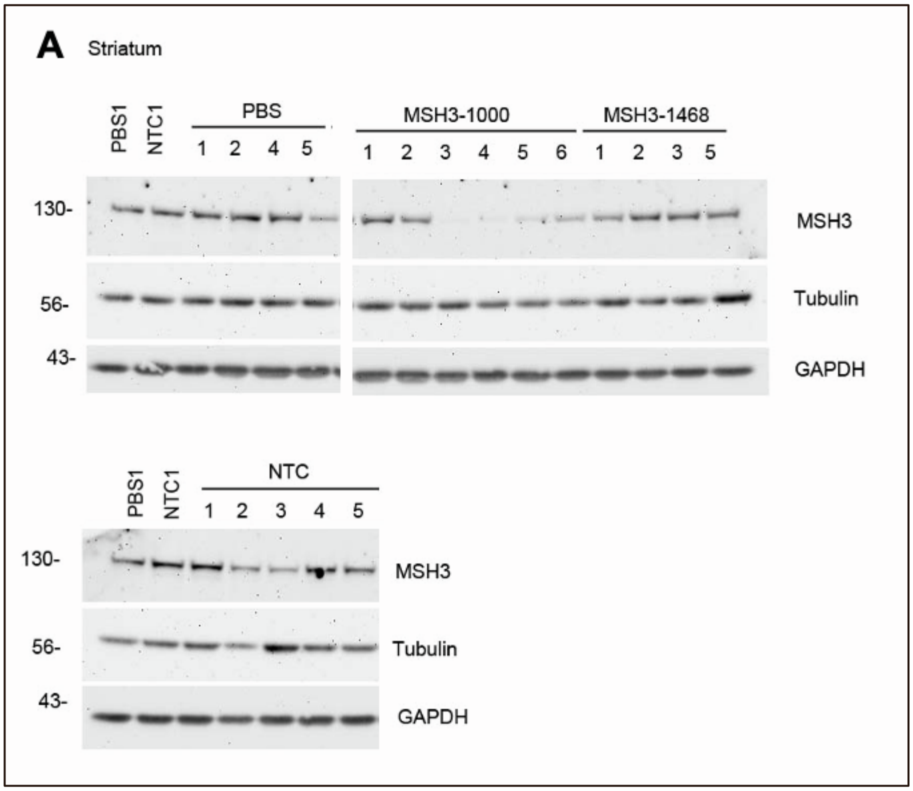

Figure S6: MSH3 targeting siRNA induce silencing *in vivo* four months post ICV injection in BAC-CAG HD mice. Western blots used to quantify MSH3 and HTT protein expression in **(A)** striatum. MSH3 expression normalized to GAPDH loading control. Tubulin used as an additional control. Treatment group listed above each section. Molecular weight of target band reported on left. Western blot conditions reported in methods.

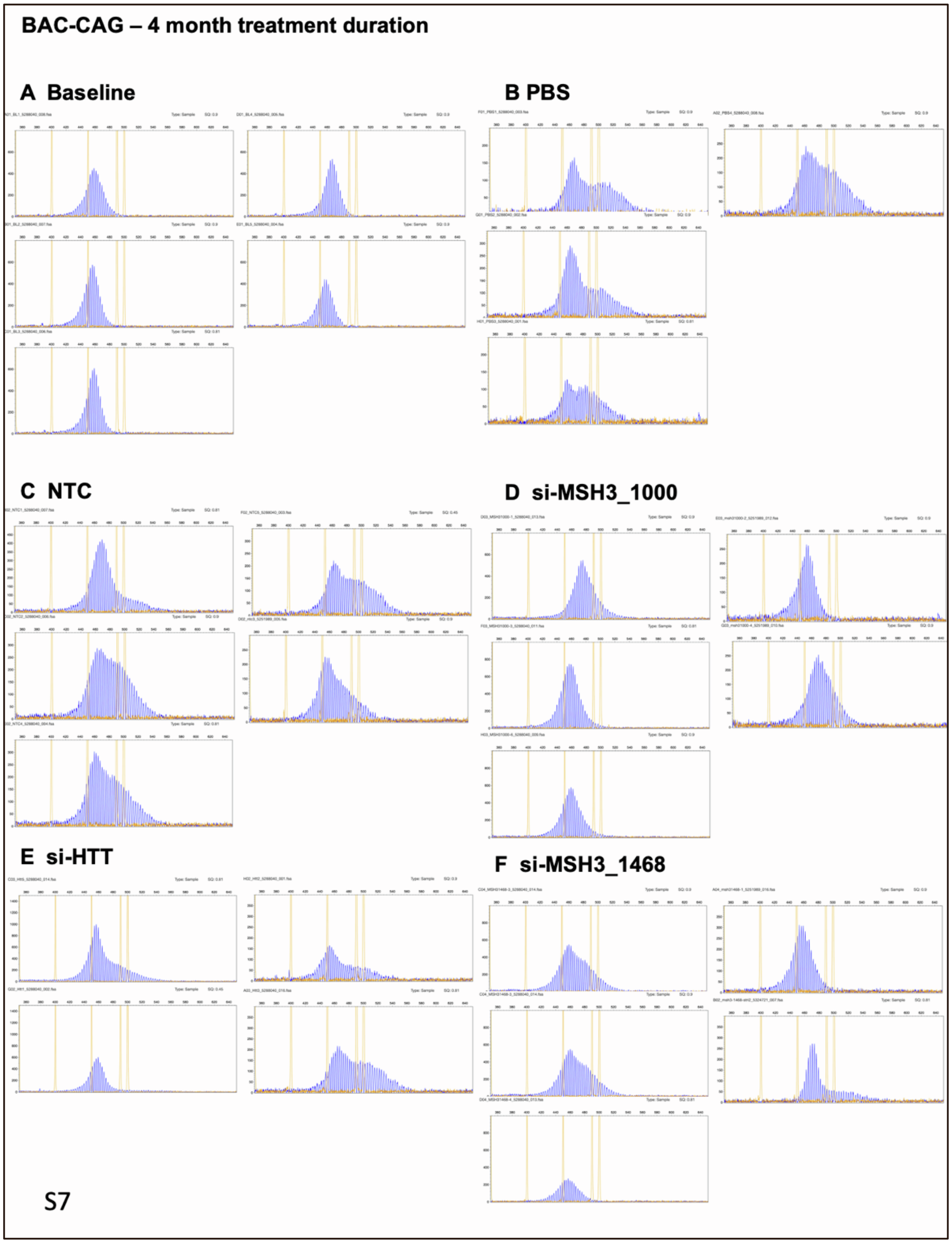

Figure S7: Fragment analysis traces indicate that siMSH3 blocks somatic repeat expansion in BAC-CAG following four-month treatment. Fragment analysis traces used to quantify somatic repeat expansion instability index for (A) Baseline, (B) PBS, (C) NTC, (D) siMSH3_1000, (E) si-Htt, (F) siMSH3_1468. X-axis of each chart is fragment length (base pairs). Y-axis is the relative fluorescence units normalized to region of interest height. Orange lines are fragment ladders.

Table S1: Summary of siRNA chemistries used for *in vitro* and *in vivo* studies

Compound	Guide strand chemistry (5'-3')	Passenger strand chemistry (5'-3')
MSH3_885	P(mU)#(fG)#(mG)(fC)(fA)(fA)(mU)(fA)(mA)(fA)(mU)(fA)(mU)#(fU)#(mG)#(fA)#(mG)#(mC)#(mU)#(fC)	(mC)#(mA)#(fA)(mU)(fA)(mU)(fU)(mU)(fA)(mU)(mU)(mG)(fC)#(mC)#(mA)-TegChol
MSH3_993	P(mU)#(fC)#(mA)(fG)(fU)(fU)(mU)(fG)(mC)(fU)(mU)(fC)(mA)#(fC)#(mA)#(fA)#(mC)#(mU)#(mC)#(fC)	(mU)#(mG)#(fU)(mG)(fA)(mA)(fG)(mC)(fA)(mA)(mA)(mC)(fU)#(mG)#(mA)-TegChol
MSH3_1000	P(mU)#(fG)#(mC)(fA)(fG)(fU)(mU)(fU)(mC)(fA)(mG)(fU)(mU)#(fU)#(mG)#(fC)#(mU)#(mU)#(mC)#(fA)	(mC)#(mA)#(fA)(mA)(fC)(mU)(fG)(mA)(fA)(mA)(mC)(mU)(fG)#(mC)#(mA)-TegChol
MSH3_1468	P(mU)#(fA)#(mA)(fA)(fG)(fC)(mA)(fU)(mG)(fG)(mC)(fU)(mG)#(fU)#(mA)#(fU)#(mU)#(mC)#(mA)#(fA)	(mU)#(mA)#(fC)(mA)(fG)(mC)(fC)(mA)(fU)(mG)(mC)(mU)(fU)#(mU)#(mA)-TegChol
MSH3_1763	P(mU)#(fA)#(mG)(fU)(fG)(fU)(mG)(fG)(mU)(fC)(mU)(fA)(mA)#(fA)#(mA)#(fC)#(mC)#(mC)#(mA)#(fC)	(mU)#(mU)#(fU)(mU)(fA)(mG)(fA)(mC)(fC)(mA)(mC)(mA)(fC)#(mU)#(mA)-TegChol
MSH3_1953	P(mU)#(fU)#(mG)(fU)(fG)(fA)(mU)(fA)(mA)(fA)(mU)(fG)(mC)#(fU)#(mA)#(fC)#(mA)#(mG)#(mA)#(fG)	(mU)#(mA)#(fG)(mC)(fA)(mU)(fU)(mU)(fA)(mU)(mC)(mA)(fC)#(mA)#(mA)-TegChol
MSH3_2048	P(mU)#(fG)#(mU)(fG)(fG)(fG)(mA)(fA)(mU)(fU)(mA)(fA)(mC)#(fA)#(mG)#(fC)#(mA)#(mG)#(mG)#(fU)	(mC)#(mU)#(fG)(mU)(fU)(mA)(fA)(mU)(fU)(mC)(mC)(mC)(fA)#(mC)#(mA)-TegChol
MSH3_2170	P(mU)#(fA)#(mA)(fU)(fA)(fA)(mU)(fU)(mC)(fA)(mG)(fU)(mU)#(fU)#(mU)#(fA)#(mU)#(mC)#(mC)#(fC)	(mA)#(mA)#(fA)(mA)(fC)(mU)(fG)(mA)(fA)(mU)(mU)(mA)(fU)#(mU)#(mA)-TegChol
MSH3_2204	P(mU)#(fC)#(mU)(fU)(fU)(fU)(mU)(fU)(mA)(fU)(mU)(fA)(mA)#(fA)#(mG)#(fG)#(mG)#(mA)#(mA)#(fG)	(mC)#(mU)#(fU)(mU)(fA)(mA)(fU)(mA)(fA)(mA)(mA)(mA)(fA)#(mG)#(mA)-TegChol
MSH3_2397	P(mU)#(fG)#(mG)(fC)(fU)(fC)(mA)(fC)(mA)(fG)(mC)(fU)(mU)#(fU)#(mU)#(fG)#(mU)#(mG)#(mC)#(fU)	(mA)#(mA)#(fA)(mA)(fG)(mC)(fU)(mG)(fU)(mG)(mA)(mG)(fC)#(mC)#(mA)-TegChol
MSH3_2675	P(mU)#(fC)#(mA)(fG)(fC)(fA)(mA)(fC)(mA)(fC)(mA)(fU)(mC)#(fA)#(mA)#(fU)#(mC)#(mA)#(mC)#(fA)	(mU)#(mU)#(fG)(mA)(fU)(mG)(fU)(mG)(fU)(mU)(mG)(mC)(fU)#(mG)#(mA)-TegChol
MSH3_2897	P(mU)#(fU)#(mA)(fU)(fA)(fU)(mU)(fG)(mU)(fC)(mU)(fG)(mC)#(fA)#(mG)#(fC)#(mA)#(mC)#(mC)#(fC)	(mC)#(mU)#(fG)(mC)(fA)(mG)(fA)(mC)(fA)(mA)(mU)(mA)(fU)#(mA)#(mA)-TegChol
MSH3_566	P(mU)#(fA)#(mU)(fC)(fA)(fG)(mU)(fA)(mC)(fA)(mU)(fU)(mU)(fU)#(mG)#(fG)#(mC)#(mA)#(mG)#(fA)#(mU)	(mC)#(mC)#(mA)(fA)(mA)(fA)(mU)(fG)(mU)(fA)(mC)(mU)(mG)(fA)#(mU)#(mA)-TegChol
MSH3_672	P(mU)#(fC)#(mA)(fA)(fA)(fC)(mU)(fG)(mA)(fC)(mU)(fG)(mA)(fG)#(mA)#(fU)#(mC)#(mA)#(mA)#(fA)#(mU)	(mA)#(mU)#(mC)(fU)(mC)(fA)(mG)(fU)(mC)(fA)(mG)(mU)(mU)(fU)#(mG)#(mA)-TegChol
MSH3_715	P(mU)#(fG)#(mA)(fA)(fG)(fC)(mA)(fG)(mU)(fU)(mU)(fU)(mC)(fU)#(mG)#(fU)#(mA)#(mA)#(mA)#(fU)#(mU)	(mA)#(mC)#(mA)(fG)(mA)(fA)(mA)(fA)(mC)(fU)(mG)(mC)(mU)(fU)#(mC)#(mA)-TegChol
MSH3_1452	P(mU)#(fC)#(mA)(fA)(fA)(fA)(mU)(fA)(mA)(fA)(mU)(fG)(mU)(fU)#(mA)#(fU)#(mC)#(mC)#(mA)#(fU)#(mU)	(mA)#(mU)#(mA)(fA)(mC)(fA)(mU)(fU)(mU)(fA)(mU)(mU)(mU)(fU)#(mG)#(mA)-TegChol
MSH3_1487	P(mU)#(fA)#(mA)(fA)(fC)(fU)(mC)(fU)(mG)(fU)(mA)(fA)(mC)(fU)#(mG)#(fC)#(mC)#(mU)#(mG)#(fG)#(mU)	(mG)#(mC)#(mA)(fG)(mU)(fU)(mA)(fC)(mA)(fG)(mA)(mG)(mU)(fU)#(mU)#(mA)-TegChol
MSH3_1505	P(mU)#(fA)#(mA)(fC)(fU)(fG)(mU)(fA)(mU)(fC)(mU)(fU)(mU)(fU)#(mG)#(fC)#(mA)#(mU)#(mA)#(fA)#(mU)	(mG)#(mC)#(mA)(fA)(mA)(fA)(mG)(fA)(mU)(fA)(mC)(mA)(mG)(fU)#(mU)#(mA)-TegChol
MSH3_1521	P(mU)#(fG)#(mA)(fG)(fA)(fA)(mC)(fC)(mU)(fU)(mU)(fG)(mA)(fU)#(mG)#(fU)#(mC)#(mA)#(mA)#(fC)#(mU)	(mA)#(mC)#(mA)(fU)(mC)(fA)(mA)(fA)(mG)(fG)(mU)(mU)(mC)(fU)#(mC)#(mA)-TegChol
MSH3_1548	P(mU)#(fC)#(mU)(fA)(fA)(fG)(mU)(fU)(mA)(fA)(mC)(fA)(mA)(fU)#(mG)#(fC)#(mC)#(mA)#(mG)#(fA)#(mU)	(mG)#(mC)#(mA)(fU)(mU)(fG)(mU)(fU)(mA)(fA)(mC)(mU)(mU)(fA)#(mG)#(mA)-TegChol
MSH3_1590	P(mU)#(fG)#(mG)(fU)(fA)(fU)(mU)(fU)(mU)(fA)(mU)(fG)(mA)(fU)#(mG)#(fG)#(mC)#(mA)#(mG)#(fC)#(mU)	(mC)#(mC)#(mA)(fU)(mC)(fA)(mU)(fA)(mA)(fA)(mA)(mU)(mA)(fC)#(mC)#(mA)-TegChol
MSH3_1647	P(mU)#(fA)#(mU)(fA)(fG)(fC)(mU)(fG)(mU)(fU)(mU)(fA)(mA)(fA)#(mA)#(fU)#(mU)#(mC)#(mU)#(fC)#(mU)	(mA)#(mU)#(mU)(fU)(mU)(fA)(mA)(fA)(mC)(fA)(mG)(mC)(mU)(fA)#(mU)#(mA)-TegChol
MSH3_1654	P(mU)#(fU)#(mU)(fA)(fC)(fU)(mU)(fG)(mA)(fU)(mA)(fG)(mC)(fU)#(mG)#(fU)#(mU)#(mU)#(mA)#(fA)#(mU)	(mA)#(mC)#(mA)(fG)(mC)(fU)(mA)(fU)(mC)(fA)(mA)(mG)(mU)(fA)#(mA)#(mA)-TegChol
MSH3_1665	P(mU)#(fU)#(mA)(fA)(fA)(fU)(mU)(fC)(mC)(fA)(mU)(fU)(mU)(fU)#(mA)#(fC)#(mU)#(mU)#(mG)#(fA)#(mU)	(mG)#(mU)#(mA)(fA)(mA)(fA)(mU)(fG)(mG)(fA)(mA)(mU)(mU)(fU)#(mA)#(mA)-TegChol
MSH3_1666	P(mU)#(fA)#(mU)(fA)(fA)(fA)(mU)(fU)(mC)(fC)(mA)(fU)(mU)(fU)#(mU)#(fA)#(mC)#(mU)#(mU)#(fG)#(mU)	(mU)#(mA)#(mA)(fA)(mA)(fU)(mG)(fG)(mA)(fA)(mU)(mU)(mU)(fA)#(mU)#(mA)-TegChol
MSH3_1675	P(mU)#(fU)#(mU)(fA)(fA)(fU)(mU)(fG)(mU)(fC)(mA)(fU)(mA)(fA)#(mA)#(fU)#(mU)#(mC)#(mC)#(fA)#(mU)	(mA)#(mU)#(mU)(fU)(mA)(fU)(mG)(fA)(mC)(fA)(mA)(mU)(mU)(fA)#(mA)#(mA)-TegChol
MSH3_1697	P(mU)#(fC)#(mA)(fG)(fA)(fU)(mU)(fC)(mC)(fU)(mU)(fA)(mA)(fU)#(mG)#(fU)#(mU)#(mG)#(mU)#(fU)#(mU)	(mA)#(mC)#(mA)(fU)(mU)(fA)(mA)(fG)(mG)(fA)(mA)(mU)(mC)(fU)#(mG)#(mA)-TegChol
MSH3_1903	P(mU)#(fU)#(mG)(fA)(fU)(fU)(mU)(fU)(mC)(fU)(mA)(fU)(mC)(fU)#(mG)#(fA)#(mC)#(mC)#(mA)#(fA)#(mU)	(mU)#(mC)#(mA)(fG)(mA)(fU)(mA)(fG)(mA)(fA)(mA)(mA)(mU)(fC)#(mA)#(mA)-TegChol
MSH3_1905	P(mU)#(fG)#(mA)(fU)(fG)(fA)(mU)(fU)(mU)(fU)(mC)(fU)(mA)(fU)#(mC)#(fU)#(mG)#(mA)#(mC)#(fC)#(mU)	(mA)#(mG)#(mA)(fU)(mA)(fG)(mA)(fA)(mA)(fA)(mU)(mC)(mA)(fU)#(mC)#(mA)-TegChol
MSH3_1980	P(mU)#(fA)#(mG)(fA)(fA)(fG)(mA)(fA)(mC)(fU)(mC)(fU)(mU)(fG)#(mG)#(fG)#(mU)#(mA)#(mG)#(fA)#(mU)	(mC)#(mC)#(mC)(fA)(mA)(fG)(mA)(fG)(mU)(fU)(mC)(mU)(mU)(fC)#(mU)#(mA)-TegChol
MSH3_2019	P(mU)#(fG)#(mA)(fA)(fA)(fU)(mU)(fC)(mU)(fG)(mA)(fC)(mU)(fU)#(mU)#(fA)#(mG)#(mG)#(mU)#(fG)#(mU)	(mU)#(mA)#(mA)(fA)(mG)(fU)(mC)(fA)(mG)(fA)(mA)(mU)(mU)(fU)#(mC)#(mA)-TegChol
MSH3_2036	P(mU)#(fA)#(mG)(fC)(fA)(fG)(mG)(fU)(mA)(fU)(mU)(fA)(mU)(fU)#(mG)#(fC)#(mU)#(mU)#(mG)#(fA)#(mU)	(mG)#(mC)#(mA)(fA)(mU)(fA)(mA)(fU)(mA)(fC)(mC)(mU)(mG)(fC)#(mU)#(mA)-TegChol

MSH3_2190	P(mU)#(fG)#(mG)(fA)(fA)(fG)(mU)(fC)(mA)(fG)(mA)(fA)(mA)(fG)#(mG)#(fU)#(mC)#(mU)#(mU)#(fU)#(mU)	(mA)#(mC)#(mC)(fU)(mU)(fU)(mC)(fU)(mG)(fA)(mC)(mU)(mU)(fC)#(mC)#(mA)-TegChol
MSH3_2199	P(mU)#(fU)#(mU)(fA)(fU)(fU)(mA)(fA)(mA)(fG)(mG)(fG)(mA)(fA)#(mG)#(fU)#(mC)#(mA)#(mG)#(fA)#(mU)	(mA)#(mC)#(mU)(fU)(mC)(fC)(mC)(fU)(mU)(fU)(mA)(mA)(mU)(fA)#(mA)#(mA)-TegChol
MSH3_2339	P(mU)#(fA)#(mG)(fA)(fG)(fU)(mU)(fC)(mU)(fU)(mU)(fA)(mU)(fU)#(mU)#(fC)#(mU)#(mA)#(mU)#(fC)#(mU)	(mG)#(mA)#(mA)(fA)(mU)(fA)(mA)(fA)(mG)(fA)(mA)(mC)(mU)(fC)#(mU)#(mA)-TegChol
MSH3_2713	P(mU)#(fA)#(mA)(fA)(fU)(fC)(mU)(fG)(mU)(fA)(mU)(fU)(mA)(fU)#(mU)#(fU)#(mG)#(mG)#(mG)#(fA)#(mU)	(mA)#(mA)#(mA)(fU)(mA)(fA)(mU)(fA)(mC)(fA)(mG)(mA)(mU)(fU)#(mU)#(mA)-TegChol
MSH3_2790	P(mU)#(fC)#(mA)(fA)(fC)(fU)(mU)(fG)(mU)(fU)(mU)(fU)(mA)(fU)#(mG)#(fU)#(mA)#(mG)#(mG)#(fA)#(mU)	(mA)#(mC)#(mA)(fU)(mA)(fA)(mA)(fA)(mC)(fA)(mA)(mG)(mU)(fU)#(mG)#(mA)-TegChol
MSH3_2975	P(mU)#(fC)#(mA)(fA)(fG)(fG)(mA)(fC)(mU)(fG)(mU)(fG)(mA)(fU)#(mG)#(fU)#(mU)#(mG)#(mC)#(fU)#(mU)	(mA)#(mC)#(mA)(fU)(mC)(fA)(mC)(fA)(mG)(fU)(mC)(mC)(mU)(fU)#(mG)#(mA)-TegChol
MSH3_3080	P(mU)#(fC)#(mA)(fG)(fG)(fG)(mU)(fU)(mA)(fA)(mG)(fG)(mA)(fU)#(mU)#(fU)#(mC)#(mA)#(mC)#(fA)#(mU)	(mA)#(mA)#(mA)(fU)(mC)(fC)(mU)(fU)(mA)(fA)(mC)(mC)(mC)(fU)#(mG)#(mA)-TegChol
MSH3_3261	P(mU)#(fU)#(mC)(fC)(fU)(fU)(mG)(fC)(mU)(fG)(mC)(fA)(mA)(fU)#(mU)#(fC)#(mC)#(mU)#(mC)#(fU)#(mU)	(mG)#(mA)#(mA)(fU)(mU)(fG)(mC)(fA)(mG)(fC)(mA)(mA)(mG)(fG)#(mA)#(mA)-TegChol
MSH3_3270	P(mU)#(fA)#(mU)(fC)(fC)(fA)(mU)(fA)(mA)(fC)(mU)(fC)(mC)(fU)#(mU)#(fG)#(mC)#(mU)#(mG)#(fC)#(mU)	(mC)#(mA)#(mA)(fG)(mG)(fA)(mG)(fU)(mU)(fA)(mU)(mG)(mG)(fA)#(mU)#(mA)-TegChol
MSH3_3401	P(mU)#(fC)#(mA)(fU)(fC)(fG)(mU)(fC)(mC)(fA)(mU)(fA)(mA)(fC)#(mU)#(fU)#(mU)#(mG)#(mC)#(fA)#(mU)	(mA)#(mA)#(mG)(fU)(mU)(fA)(mU)(fG)(mG)(fA)(mC)(mG)(mA)(fU)#(mG)#(mA)-TegChol
MSH3_3464	P(mU)#(fA)#(mG)(fA)(fA)(fG)(mU)(fC)(mU)(fG)(mU)(fG)(mU)(fU)#(mU)#(fC)#(mU)#(mU)#(mC)#(fC)#(mU)	(mG)#(mA)#(mA)(fA)(mC)(fA)(mC)(fA)(mG)(fA)(mC)(mU)(mU)(fC)#(mU)#(mA)-TegChol
MSH3_3559	P(mU)#(fA)#(mA)(fG)(fA)(fU)(mA)(fG)(mG)(fC)(mU)(fG)(mU)(fU)#(mA)#(fC)#(mU)#(mG)#(mG)#(fA)#(mU)	(mG)#(mU)#(mA)(fA)(mC)(fA)(mG)(fC)(mC)(fU)(mA)(mU)(mC)(fU)#(mU)#(mA)-TegChol
MSH3_3588	P(mU)#(fG)#(mU)(fC)(fA)(fU)(mA)(fA)(mU)(fU)(mU)(fU)(mA)(fU)#(mG)#(fC)#(mU)#(mC)#(mA)#(fC)#(mU)	(mG)#(mC)#(mA)(fU)(mA)(fA)(mA)(fA)(mU)(fU)(mA)(mU)(mG)(fA)#(mC)#(mA)-TegChol
MSH3_3621	P(mU)#(fA)#(mA)(fA)(fC)(fC)(mU)(fC)(mU)(fC)(mU)(fG)(mU)(fU)#(mU)#(fC)#(mC)#(mA)#(mA)#(fU)#(mU)	(mG)#(mA)#(mA)(fA)(mC)(fA)(mG)(fA)(mG)(fA)(mG)(mG)(mU)(fU)#(mU)#(mA)-TegChol
MSH3_3694	P(mU)#(fG)#(mG)(fA)(fA)(fG)(mU)(fC)(mU)(fA)(mU)(fU)(mC)(fA)#(mA)#(fG)#(mA)#(mG)#(mU)#(fG)#(mU)	(mC)#(mU)#(mU)(fG)(mA)(fA)(mU)(fA)(mG)(fA)(mC)(mU)(mU)(fC)#(mC)#(mA)-TegChol
MSH3_3715	P(mU)#(fA)#(mA)(fA)(fA)(fU)(mU)(fU)(mU)(fC)(mU)(fA)(mA)(fU)#(mU)#(fA)#(mC)#(mA)#(mA)#(fA)#(mU)	(mU)#(mA)#(mA)(fU)(mU)(fA)(mG)(fA)(mA)(fA)(mA)(mU)(mU)(fU)#(mU)#(mA)-TegChol
MSH3_3761	P(mU)#(fG)#(mG)(fA)(fA)(fU)(mU)(fA)(mU)(fA)(mU)(fU)(mC)(fU)#(mG)#(fC)#(mC)#(mA)#(mC)#(fU)#(mU)	(mG)#(mC)#(mA)(fG)(mA)(fA)(mU)(fA)(mU)(fA)(mA)(mU)(mU)(fC)#(mC)#(mA)-TegChol
MSH3_3818	P(mU)#(fU)#(mG)(fA)(fA)(fC)(mU)(fG)(mA)(fA)(mA)(fC)(mA)(fA)#(mA)#(fU)#(mA)#(mA)#(mA)#(fA)#(mU)	(mA)#(mU)#(mU)(fU)(mG)(fU)(mU)(fU)(mC)(fA)(mG)(mU)(mU)(fC)#(mA)#(mA)-TegChol
MSH3_3870	P(mU)#(fA)#(mU)(fU)(fU)(fU)(mA)(fG)(mU)(fU)(mC)(fA)(mA)(fU)#(mG)#(fG)#(mA)#(mU)#(mA)#(fG)#(mU)	(mC)#(mC)#(mA)(fU)(mU)(fG)(mA)(fA)(mC)(fU)(mA)(mA)(mA)(fA)#(mU)#(mA)-TegChol
MSH3_3876	P(mU)#(fA)#(mA)(fA)(fA)(fU)(mU)(fA)(mU)(fU)(mU)(fU)(mA)(fG)#(mU)#(fU)#(mC)#(mA)#(mA)#(fU)#(mU)	(mA)#(mA)#(mC)(fU)(mA)(fA)(mA)(fA)(mU)(fA)(mA)(mU)(mU)(fU)#(mU)#(mA)-TegChol
MSH3_4283	P(mU)#(fU)#(mU)(fG)(fA)(fU)(mA)(fA)(mU)(fU)(mC)(fU)(mA)(fU)#(mU)#(fU)#(mC)#(mU)#(mU)#(fU)#(mU)	(mA)#(mA)#(mA)(fU)(mA)(fG)(mA)(fA)(mU)(fU)(mA)(mU)(mC)(fA)#(mA)#(mA)-TegChol
MSH3_4315	P(mU)#(fC)#(mU)(fU)(fA)(fU)(mU)(fC)(mC)(fU)(mU)(fC)(mU)(fG)#(mU)#(fG)#(mC)#(mU)#(mC)#(fU)#(mU)	(mC)#(mA)#(mC)(fA)(mG)(fA)(mA)(fG)(mG)(fA)(mA)(mU)(mA)(fA)#(mG)#(mA)-TegChol
MSH3_4320	P(mU)#(fA)#(mU)(fG)(fA)(fC)(mC)(fU)(mU)(fA)(mU)(fU)(mC)(fC)#(mU)#(fU)#(mC)#(mU)#(mG)#(fU)#(mU)	(mA)#(mA)#(mG)(fG)(mA)(fA)(mU)(fA)(mA)(fG)(mG)(mU)(mC)(fA)#(mU)#(mA)-TegChol
MSH3_4336	P(mU)#(fU)#(mA)(fA)(fC)(fC)(mU)(fU)(mU)(fU)(mA)(fA)(mA)(fU)#(mU)#(fU)#(mC)#(mA)#(mU)#(fG)#(mU)	(mA)#(mA)#(mA)(fU)(mU)(fU)(mA)(fA)(mA)(fA)(mG)(mG)(mU)(fU)#(mA)#(mA)-TegChol
MSH3_4371	P(mU)#(fA)#(mC)(fA)(fA)(fU)(mC)(fU)(mU)(fU)(mA)(fA)(mA)(fC)#(mU)#(fG)#(mC)#(mU)#(mU)#(fA)#(mU)	(mC)#(mA)#(mG)(fU)(mU)(fU)(mA)(fA)(mA)(fG)(mA)(mU)(mU)(fG)#(mU)#(mA)-TegChol
MSH3_4372	P(mU)#(fA)#(mA)(fC)(fA)(fA)(mU)(fC)(mU)(fU)(mU)(fA)(mA)(fA)#(mC)#(fU)#(mG)#(mC)#(mU)#(fU)#(mU)	(mA)#(mG)#(mU)(fU)(mU)(fA)(mA)(fA)(mG)(fA)(mU)(mU)(mG)(fU)#(mU)#(mA)-TegChol
MSH3_4379	P(mU)#(fU)#(mU)(fC)(fA)(fU)(mC)(fC)(mA)(fA)(mC)(fA)(mA)(fU)#(mC)#(fU)#(mU)#(mU)#(mA)#(fA)#(mU)	(mA)#(mG)#(mA)(fU)(mU)(fG)(mU)(fU)(mG)(fG)(mA)(mU)(mG)(fA)#(mA)#(mA)-TegChol
MSH3_4410	P(mU)#(fA)#(mU)(fA)(fU)(fU)(mU)(fA)(mU)(fU)(mA)(fC)(mU)(fU)#(mG)#(fA)#(mA)#(mU)#(mG)#(fA)#(mU)	(mU)#(mC)#(mA)(fA)(mG)(fU)(mA)(fA)(mU)(fA)(mA)(mA)(mU)(fA)#(mU)#(mA)-TegChol
NTC_P3_*in vitro*	P(mU)#(fA)#(mA)(fU)(fC)(fG)(mU)(fA)(mU)(fU)(mU)(fG)(mU)#(fC)#(mA)#(fA)#(mU)#(mC)#(fA)#(mU)	(mG)#(mA)#(mU)(mU)(mG)(fA)(mC)(fA)(mA)(fA)(mU)(fA)(mC)(mG)(mA)(fU)#(mU)#(mA)-TegChol
MSH3_1000_*in vivo*	V(mU)#(fG)#(mC)(fA)(fG)(fU)(mU)(fU)(mC)(fA)(mG)(fU)(mU)(fU)#(mG)#(fC)#(mU)#(mU)#(mC)#(fA)#(mU)	(mG)#(mC)#(mA)(fA)(mA)(fC)(mU)(fG)(mA)(fA)(mA)(mC)(mU)(fG)#(mC)#(mA)-DIO
HTT_10150_*in vivo*	V(mU)#(fU)#(mA)(fA)(fU)(fC)(mU)(fC)(mU)(fU)(mU)(fA)(mC)(fU)#(mG)#(fA)#(mU)#(mA)#(mU)#(fA)#(mU)	(mU)#(mC)#(mA)(fG)(mU)(fA)(mA)(fA)(mG)(fA)(mG)(mA)(mU)(fU)#(mA)#(mA)-DIO
NTC_P3_AS_21mer	V(mU)#(fA)#(mA)(fU)(fC)(fG)(mU)(fA)(mU)(fU)(mU)(fG)(mU)(fC)#(mA)#(fA)#(mU)#(mC)#(mA)#(fU)#(mU)	(mU)#(mU)#(mG)(fA)(mC)(fA)(mA)(fA)(mU)(fA)(mC)(mG)(mA)(fU)#(mU)#(mA)-DIO
HTT_10150_*in vivo 2*	V(mU)#(fU)#(mA)(fA)(fU)(fC)(mU)(fC)(mU)(fU)(mU)(fA)(mC)(fU)#(mG)#(fA)#(mU)#(mA)#(mU)#(mA)#(fU)	(mU)#(mC)#(mA)(fG)(mU)(fA)(mA)(fA)(mG)(fA)(mG)(mA)(mU)(fU)#(mA)#(mA)-DIO
NTC_P3_AS_21mer	V(mU)#(fA)#(mA)(fU)(fC)(fG)(mU)(fA)(mU)(fU)(mU)(fG)(mU)(fC)#(mA)#(fA)#(mU)#(mC)#(mA)#(mU)#(fU)	(mU)#(mU)#(mG)(fA)(mC)(fA)(mA)(fA)(mU)(fA)(mC)(mG)(mA)(fU)#(mU)#(mA)-DIO

Table S2: Summary of Mouse Strain, Genotype and CAG level

Please see the attached excel document for the full list of genotypes and mouse strain information
