# Supplementary material for: Di-valent siRNA-mediated silencing of MSH3 blocks somatic repeat expansion in mouse models of Huntington’s disease
Source: Mol Ther. 2023 May 12;31(6):1661–74. doi: 10.1016/j.ymthe.2023.05.006 (PMC10277892; doi:10.1016/j.ymthe.2023.05.006)
Supplement: Document S2. Article plus supplemental information [file mmc3.pdf]

Molecular Therapy
Original Article

Di-valent siRNA-mediated silencing of MSH3 blocks somatic repeat expansion in mouse models of Huntington's disease

Daniel O'Reilly,[1,8] Jillian Belgrad,[1,8] Chantal Ferguson,[1] Ashley Summers,[1] Ellen Sapp,[2] Cassandra McHugh,[5] Ella Mathews,[5] Adel Boudi,[2] Julianna Buchwald,[1] Socheata Ly,[1] Dimas Moreno,[1] Raymond Furgal,[1] Eric Luu,[1] Zachary Kennedy,[1] Vignesh Hariharan,[1] Kathryn Monopoli,[1] X. William Yang,[3,4] Jeffery Carroll,[5,6] Marian DiFiglia,[2] Neil Aronin,[1,7] and Anastasia Khvorova[1]

[1]RNA Therapeutics Institute, University of Massachusetts Chan Medical School, Worcester, MA 01605, USA; [2]Department of Neurology, Massachusetts General Hospital, Boston, MA 02114, USA; [3]Center for Neurobehavioral Genetics, Jane and Terry Semel Institute of Neuroscience and Human Behavior, University of California Los Angeles, Los Angeles, CA 90095, USA; [4]Department of Psychiatry and Biobehavioral Sciences, David Geffen School of Medicine, University of California Los Angeles, Los Angeles, CA 90095, USA; [5]Behavioral Neuroscience Program, Psychology Department, Western Washington University, Bellingham, WA 98225, USA; [6]Department of Neurology, University of Washington, Seattle, WA 98104-2499, USA; [7]Department of Medicine, University of Massachusetts Chan Medical School, Worcester, MA 01605, USA

Huntington's disease (HD) is a severe neurodegenerative disorder caused by the expansion of the CAG trinucleotide repeat tract in the huntingtin gene. Inheritance of expanded CAG repeats is needed for HD manifestation, but further somatic expansion of the repeat tract in non-dividing cells, particularly striatal neurons, hastens disease onset. Called somatic repeat expansion, this process is mediated by the mismatch repair (MMR) pathway. Among MMR components identified as modifiers of HD onset, MutS homolog 3 (MSH3) has emerged as a potentially safe and effective target for therapeutic intervention. Here, we identify a fully chemically modified short interfering RNA (siRNA) that robustly silences Msh3 *in vitro* and *in vivo*. When synthesized in a di-valent scaffold, siRNA-mediated silencing of Msh3 effectively blocked CAG-repeat expansion in the striatum of two HD mouse models without affecting tumor-associated microsatellite instability or mRNA expression of other MMR genes. Our findings establish a promising treatment approach for patients with HD and other repeat expansion diseases.

INTRODUCTION

Huntington's disease (HD) is a rare autosomal dominant neurodegenerative disease that impairs cognitive and motor function, eventually leading to death.[1,2] Currently, no disease-modifying treatments are available.[3] HD is caused by an expansion of the CAG-repeat tract in the huntingtin gene (*HTT*), with age of disease onset being strongly driven by the number of CAG repeats.[4–6] Individuals with ≥40 CAG repeats develop HD in their 40s, whereas individuals with ≥70 repeats develop juvenile-onset HD.

CAG-repeat number is inherited, but undergoes expansion over time due to somatic instability.[7] This process, termed somatic repeat expansion, occurs preferentially in non-dividing cells with active transcription,[8,9] such as neurons, and generates significant mosaicism in patient brains.[7,10] Somatic repeat expansion occurs when repetitive DNA codons (i.e., sequential CAGs) misalign during transcription, creating a slipped-loop intermediate that recruits mismatch repair (MMR) machinery to cleave the opposite (non-slipped) strand.[11–13] The slipped loop is then used as a template to add new nucleotides that further expand the locus.[11–13]

A recent genome-wide association study identified several MMR genes as major modifiers of HD onset,[14] expansion of the CAG-repeat tract,[14,15] and clinical HD progression,[16] suggesting this pathway as a potential therapeutic target for HD. Yet, MMR is pivotal in maintaining cellular function, repairing single-base mismatches, deletions, and small and large loops to prevent genomic instability and carcinogenesis.[17–19] Mutations in MMR genes are associated with cancers, including those affecting the brain.[17,18] Thus, development of an expansion-modifying therapy for HD requires careful selection of an MMR gene target.

Among MMR gene candidates, MutS homolog 3 (MSH3) emerges as a potentially safe and effective target for knockdown. MSH3 forms a complex with MSH2, called MutSβ, that selectively recognizes large (>3 nt) DNA loops, such as those created by expanded CAG repeats, and is not involved in other pathways essential for maintenance of DNA integrity.[20] Single-nucleotide polymorphisms in the *MSH3* gene are associated with enhanced levels of CAG expansion[14,15] as well as colon cancer,[17,18] but, critically, are not associated with brain

Received 20 October 2022; accepted 8 May 2023;
https://doi.org/10.1016/j.ymthe.2023.05.006.

[8]These authors contributed equally

Correspondence: Anastasia Khvorova, RNA Therapeutics Institute, University of Massachusetts Chan Medical School, Worcester, MA 01605, USA.
E-mail: anastasia.khvorova@umassmed.edu

This is an open access article under the CC BY license (http://creativecommons.org/licenses/by/4.0/).

cancers.[21] Genetic knockout of *Msh3* blocks somatic repeat expansion in Hdh^{Q111} mice.[14,15,22,23] Exploration of pharmaceutical approaches that selectively lower MSH3 expression in the brain is warranted.

Short interfering RNA (siRNA) is a powerful therapeutic tool for sequence-specific silencing of target genes.[24,25] Whereas the siRNA sequence defines the gene target, the scaffold (i.e., pattern of chemical modifications) of an siRNA dictates stability and delivery *in vivo*.[26–28] Thus, once the scaffold of an siRNA has been optimized for delivery to a target tissue, any gene with a known sequence in that tissue can be targeted by changing the siRNA sequence.[29] This programmability streamlines discovery pipelines and enables rapid progression of compounds to the clinic. Indeed, after establishing an siRNA architecture for delivery to the liver, four siRNA drugs were rapidly developed and approved by the US FDA for treatment of liver-related conditions, with many more in late-stage clinical trials.[25,30] We recently developed an siRNA scaffold for delivery to the central nervous system (CNS), termed di-valent siRNA. By slowing clearance from the cerebrospinal fluid (CSF) and enhancing uptake into cells,[31] di-valent siRNAs support broad distribution and potent modulation of target gene expression in mouse and non-human primate (NHP) brain for up to 6 months after a single injection.[32] The placement of the CSF infusion (intrathecal or intracerebroventricular) has no significant impact on di-valent siRNA distribution in large brains, confirming clinical translatability.[33] Di-valent siRNA could allow for therapeutic modulation of MSH3 expression in the CNS, so long as a potent, fully modified siRNA sequence targeting *Msh3* can be identified.[32]

Here, we identify fully chemically stabilized siRNAs targeting human, NHP, and mouse *Msh3* and show that di-valent siRNA-mediated silencing of *Msh3* results in blockage of somatic repeat expansion over 2 and 4 months in two HD mouse models. Taken together, these results provide evidence that silencing MSH3 with siRNA is a promising therapeutic approach for HD patients.

RESULTS

Identification of potent fully chemically modified siRNA sequences that silence *Msh3* mRNA in human, mouse, and NHP cells *in vitro*

To identify therapeutic leads for MSH3 silencing, we set out to screen a panel of chemically modified siRNA sequences targeting *MSH3*. Sequences were designed using a modified siRNA efficacy prediction algorithm,[34] which scores sequences based on specificity, seed complement frequency, local structure, thermodynamic bias, G:C content, and positional base preferences.[34] A high score predicts high efficacy *in vitro* but does not estimate the level of gene silencing induced. We selected 48 high-scoring siRNA sequences with human homology and 12 high-scoring sequences with cross-homology between mice and humans (to simplify the *in vivo* validation and preclinical development path) for experimental determination of gene silencing efficacy in cells.

For the efficacy screen, siRNA compounds were synthesized in an entirely modified asymmetric scaffold[31] with an optimized 2′-O-methyl RNA/2′-fluoro RNA pattern, and all terminal backbones were phosphorothioated (Figure 1A). These chemical modifications increase siRNA potency, stability, and duration of effect *in vivo*.[27–29,35] Compounds were also modified with a 3′ cholesterol conjugate on the sense strand[26,31] to enable passive internalization into all cell types following addition to the culture medium. Sequences and chemical modification patterns of all compounds are listed in Table S1.

The entire siRNA panel (60 compounds) was screened in HeLa cells, and all 12 cross-reactive siRNAs were additionally screened in the mouse neuronal cell line N2a. HeLa cells and N2a cells are model lines for bulk screening in search of leads: they are well studied, are easy to maintain, and express human MSH3 or mouse Msh3 mRNA.[36] *MSH3* and *Msh3* mRNA levels were evaluated by QuantiGene assay at 72 h post-transfection (Figure S1). In HeLa cells, 12 human-targeting and 6 cross-reactive compounds induced >75% silencing of *MSH3* mRNA. The level of *Msh3* silencing in N2a cells was less pronounced. Indeed, several high-efficacy compounds in HeLa cells failed to induce significant silencing in N2a cells (siMSH3_1980, siMSH3_2397, siMSH3_2675). However, we did identify 5 compounds that achieved >50% silencing of *Msh3* mRNA (Figure 1B). siRNA with full sequence homology can show species-specific differences in gene silencing level and overall efficacy.[36] The observed efficacy difference between species may be driven by variability in nuclear/cytoplasmic mRNA retention and/or local changes in structural accessibility.[36,37]

The two cross-reactive compounds with the highest silencing efficacy in both human and mouse cells were siMSH3_1000 (86% in human and 75% in mouse) and siMSH3_1468 (90% in human and 77% in mouse). siMSH3_1000 (Figure 1C) and siMSH3_1468 (Figure 1D) induced dose-dependent silencing in HeLa, N2a, and LLC-MK2 NHP cell lines (IC_{50} from 15 to 479 nM).

Injection of di-valent siMSH3_1000 potently silences Msh3 and blocks somatic repeat expansion in striatum of Hdh^{Q111} mice

The heterozygous Hdh^{Q111} (C57BL/6J background) mouse model is a validated knockin model of HD in which human mutant *HTT* exon 1 is inserted into the mouse *Htt* locus.[38,39] This model possesses a 109- to 111-CAG-repeat tract that undergoes somatic repeat expansion within 2 months in the striatum.[39–41]

To test the *in vivo* efficacy of siMSH3_1000 and siMSH3_1468, each compound, along with an siRNA with a non-targeting control (NTC) sequence, were synthesized in the di-valent scaffold (Figure 2A)[32] with a 5′-vinylphosphonate to chemically stabilize the 5′ phosphate.[35,42] We also used a previously validated di-valent siRNA targeting *Htt* (siHTT_10150) as a control.[32,43] All sequence and chemical modification patterns of siRNA can be found in Table S1. Lead or control compounds (10 nmol, or 125 μg, dose) were delivered to 12-week-old Hdh^{Q111} mice (n = 6 per group) via intracerebroventricular (i.c.v.) injection (Figure 2B), and the mice were euthanized at

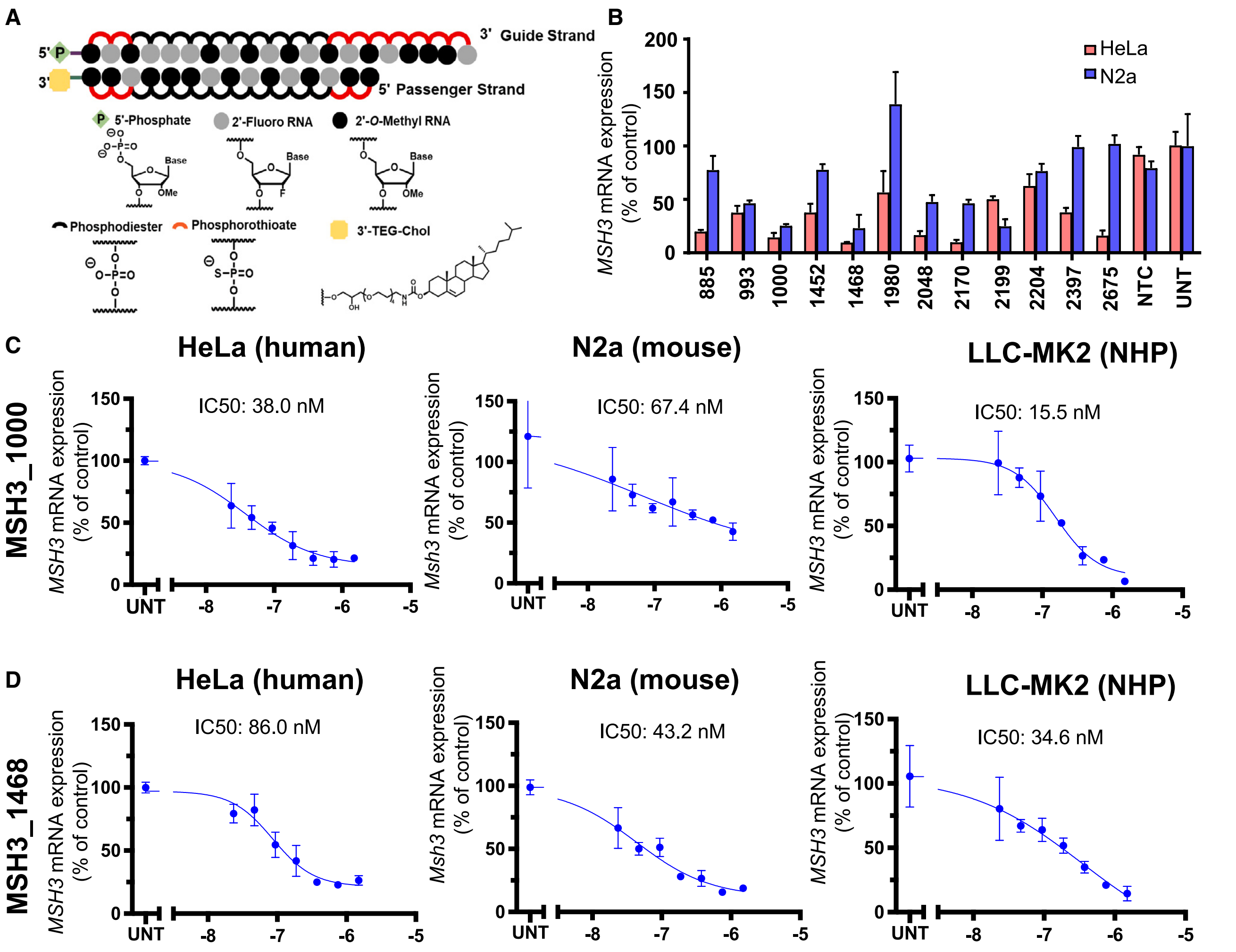

Figure 1. Silencing of MSH3 with fully chemically modified siRNA

(A) Chemical scaffold of fully modified siRNA utilized for *in vitro* screening. (B) *MSH3* mRNA was measured in HeLa (red) and Neuro2a (blue) cells 72 h post-treatment with 1.5 μM siRNA or non-targeting control (NTC). UNT denotes untreated controls. Data shown are the mean ± standard deviation. Dose-response results for (C) MSH3_1000 and (D) MSH3_1438 in HeLa (left), N2A cells (middle), and non-human primate (NHP) LLC-MK2 cells (right). Cells were treated with siRNA at the concentrations shown for 72 h. For all analyses, mRNA levels were measured using the QuantiGene Singleplex assay and calculated as the percentage of untreated.

20 weeks of age to evaluate Msh3 protein silencing and somatic repeat expansion.

At 2 months post-injection, di-valent siMSH3_1000, but not di-valent siMSH3_1468, showed potent silencing (54% silencing, $p < 0.01$) of Msh3 protein throughout the striatum (Figures 2C and S2) compared with NTC. At lower doses (62.5 and 31.3 μg), di-valent siMSH3_1000 did not induce statistically significant Msh3 silencing at 2 months post-injection. As expected, di-valent siHTT_10150 potently silenced HTT protein (>90% silencing, $p < 0.01$) in striatum (Figures 2D and S2).[32]

We measured the effects of Msh3 and HTT silencing on somatic repeat expansion at the Htt locus in the striatum using fragment analysis.[44] In each experiment, we included a group of non-injected 3-month-old littermates to determine striatal instability at the time of siRNA or control treatment. We used this measurement to confirm that sufficient expansion had occurred during the treatment window and to understand the effects of Msh3- and Htt-targeting siRNAs on this expansion. The baseline instability index for 3-month-old Hdh^{Q111} mice was 2.6 ± 1.2. The NTC- and PBS-treated groups had instability indexes of 7.1 ± 0.7 and 6.0 ± 0.7, respectively, at 2 months post-injection (Figures 2E and 2F). The significant difference in instability index between the baseline and the PBS and NTC groups indicates that the 2-month study window is adequate to detect somatic repeat expansion ($p < 0.001$). The instability indexes of the di-valent siMSH3_1000- and siMSH3_1468-treated groups (125 μg dose) 2 months post-injection were not significantly different

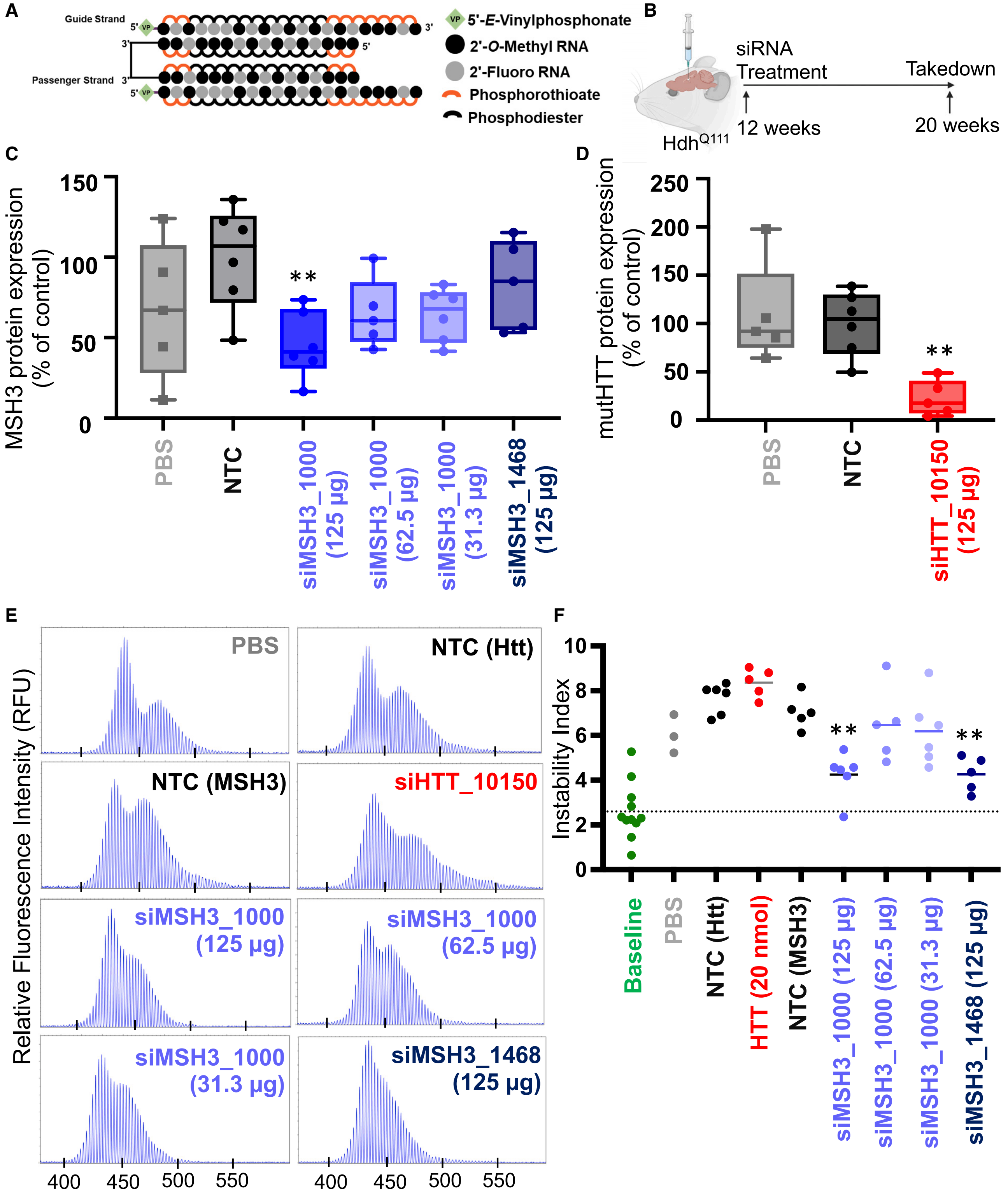

Figure 2. MSH3 silencing with di-siRNA blocks somatic repeat expansion in striatum of Hdh^{Q111} HD mice

(A) Di-valent chemically modified siRNA structure. (B) Experimental setup in Hdh^{Q111} mice depicts bilateral intracerebroventricular injection of PBS or di-siRNA targeting a non-targeting control (NTC), siHTT_10150, siMSH3_1468, or siMSH3_1000, with 125 μg siRNA per ventricle. Mice were injected at 12 weeks of age and euthanized at

(legend continued on next page)

from that of the baseline group (4.3 ± 0.9 and 4.3 ± 0.7, respectively), suggesting blockage of further expansion following treatment (Figures 2E and 2F). At lower doses of di-valent siMSH3_1000 (62.5 and 31.3 μg), we did not see statistically significant blockage of expansion (instability index of 6.5 ± 1.5 for the 62.5 μg dose and 6.2 ± 1.4 for the 31.3 μg dose). Near-complete silencing of HTT with di-valent siHTT_10150 had no measurable effect on somatic repeat expansion (instability index 8.4 ± 0.6) (Figures 2E and 2F). We also included an NTC siRNA with the exact same chemical modification pattern (aligning 2′-O-methyl RNA/2′-fluoro RNA) as divalent siHTT_10150 and observed an instability index of 7.7 ± 0.6. All traces used to calculate the instability index can be found in Figure S3.

Injection of di-valent siMSH3_1000 potently silences Msh3 and blocks somatic repeat expansion at the humanized mutant HTT locus in the BAC-CAG mouse model

To determine whether the results in the Hdh^{Q111} mouse model could be replicated in a humanized full-length mutant *HTT* context, we evaluated di-siRNA-mediated modulation of Msh3 and somatic repeat expansion in the BAC-CAG HD mouse model (Figures 3A and 3B).[45] BAC-CAG mice express a fully human mutant *HTT* gene with an ∼120- to 130-CAG-repeat tract that undergoes somatic repeat expansion over 2 months.[45] BAC-CAG is the first HD model with uninterrupted CAG repeats within the full human mutant HTT gene.[45]

Di-valent siMSH3_1000, siHTT_10150, PBS, or NTC (10 nmol, or 125 μg) was delivered to 12-week-old BAC-CAG mice via i.c.v. injection, and the mice were sacrificed at 20 weeks of age (Figure 3B). At 2 months post-injection, di-valent siHTT_10150 (Figure 3C) and siMSH3_1000 (Figure 3D) silenced HTT (70%–80% silencing vs. NTC, $p < 0.01$) and Msh3 (40%–60% silencing vs. NTC, $p < 0.05$) protein, respectively, in the striatum, cortex, and thalamus (Figure S4).

The instability index in striatum of the baseline BAC-CAG mouse group was 2.1 ± 1.5. Di-valent siMSH3_1000-treated mice maintained this index (2.3 ± 1.8), whereas NTC-treated animals had an instability index of 6.2 ± 1.2 (representative traces in Figure 3E, quantification in Figure 3F). The siHTT_10150-treated group had an instability index of 6.3 ± 1.7, suggesting no effect on somatic repeat expansion. All traces used to calculate the instability index can be found in Figure S5.

Silencing of Msh3 with siMSH3_1000 or siMSH3_1468 blocks somatic repeat expansion at 4 months in BAC-CAG HD mice

To determine if blocking somatic expansion by siRNA-mediated silencing of Msh3 is robust across siRNA sequences and time points in BAC-CAG mice, we delivered di-valent NTC, siMSH3_1000, siMSH3_1468, or PBS (10 nmol, or 125 μg) to 12-week-old BAC-CAG mice and sacrificed the mice at 28 weeks (Figures 4A and 4B). At 4 months post-injection, *Msh3* mRNA and protein silencing was 70% and 65%, respectively, in divalent siMSH3_1000-treated brain ($p < 0.001$ and $p < 0.05$, respectively; Figures 4C, 4D, and S6). *Msh3* mRNA and protein silencing was 60% and 5%, respectively, in di-valent siMSH3_1468-treated brain ($p < 0.001$ and non-significant, respectively; Figure 4D).

The somatic instability index in the baseline group was −0.50 ± 0.31. Four months post-injection, the NTC group had an instability index of 4.14 ± 1.8, whereas the di-valent siMSH3_1000-treated group had an instability index similar to baseline at 0.22 ± 0.47 (Figures 4E and 4F; $p < 0.001$, one-way ANOVA vs. NTC), suggesting blockage of further somatic instability with 60% protein reduction. Di-valent siMSH3_1468-treated striatum had an instability index of 2.68 ± 3.4 ($p < 0.05$, one-way ANOVA vs. NTC), showing reduced, but not blocked, expansion. This result suggests a potential dose-dependent relationship between Msh3 silencing and blocked somatic expansion. All traces used to calculate the instability index can be found in Figure S7.

Silencing of Msh3 has no impact on CNS microsatellite instability or mismatch repair pathway expression

Select MMR deficiency is associated with microsatellite instability in several cancers, including colon, gastric, and endometrial.[46] To investigate whether di-valent siRNA silencing of Msh3 alters CNS microsatellite instability, we probed three validated microsatellite loci from the Bethesda panel, which characterizes known unstable loci that have been identified in mouse tumors: mouse big adenine tract (mBAT) 24, mBAT 26, and mBAT 64.[47] The length of each tract was measured at 4 months in di-valent NTC- (n = 5), siMSH3_1000- (n = 5), siMSH3_1468- (n = 4), and PBS- (n = 4) treated tissue (Figure 5A). There was no measurable difference in microsatellite instability at the mBAT 24 locus ($p = 0.99$; Figures 5A and 5B), mBAT 26 locus ($p = 0.89$; Figures 5C and 5D), or mBAT 64 locus ($p = 0.99$; Figures 5E and 5F) across treatment groups (Figure 5B).

To ensure that silencing of *Msh3* had no impact on the expression of other MMR genes, we evaluated mRNA expression of *Msh2*, *Msh6*, *Mlh1*, *Mlh3*, *Pms1*, and *Pms2* in BAC-CAG brain tissue 4 months post-injection of siMSH3_1000 or NTC. We found no difference in *Msh2* ($p = 0.55$), *Msh6* ($p = 0.89$), *Mlh1* ($p = 0.56$), *Mlh3* ($p = 0.33$), *Pms1* ($p = 0.36$), or *Pms2* ($p = 0.36$) mRNA expression between groups (Figure 6). The only gene that was significantly reduced was *Msh3* itself ($p < 0.05$).

20 weeks of age. (C) MSH3 protein expression and (D) mutant Htt (mutHTT) in striatum following treatment with PBS (gray), NTC (125 μg, black), siHTT_10150 (125 μg, red), siMSH3_1000 (125, 62.5, or 31.3 μg, purple), or siMSH3_1468 (125 μg, navy). Protein expression is compared with NTC (one-way ANOVA with Dunnett's multiple comparison test, $^{**}p < 0.01$). Each data point derives from the striatum of one animal (n = 5–6 animals per condition). Data shown are the mean ± range. (E) Representative fragment analysis of the expanded CAG locus in the striatum of PBS-, NTC-, siMSH3_1000-, siMSH3_1468-, and siHTT_10150-treated Hdh^{Q111} mice, 8 weeks post-injection. Primers are reported in the materials and methods. (F) Somatic instability index calculated with a 5% signal-to-noise threshold as described in the materials and methods. Each data point is one mouse. The dotted line is the striatal instability index in 3-month-old Hdh^{Q111} mice, representing the instability at the time of injection prior to treatment. Instability index is compared with NTC (one-way ANOVA with Dunnett's multiple comparison test; $^{**}p < 0.01$).

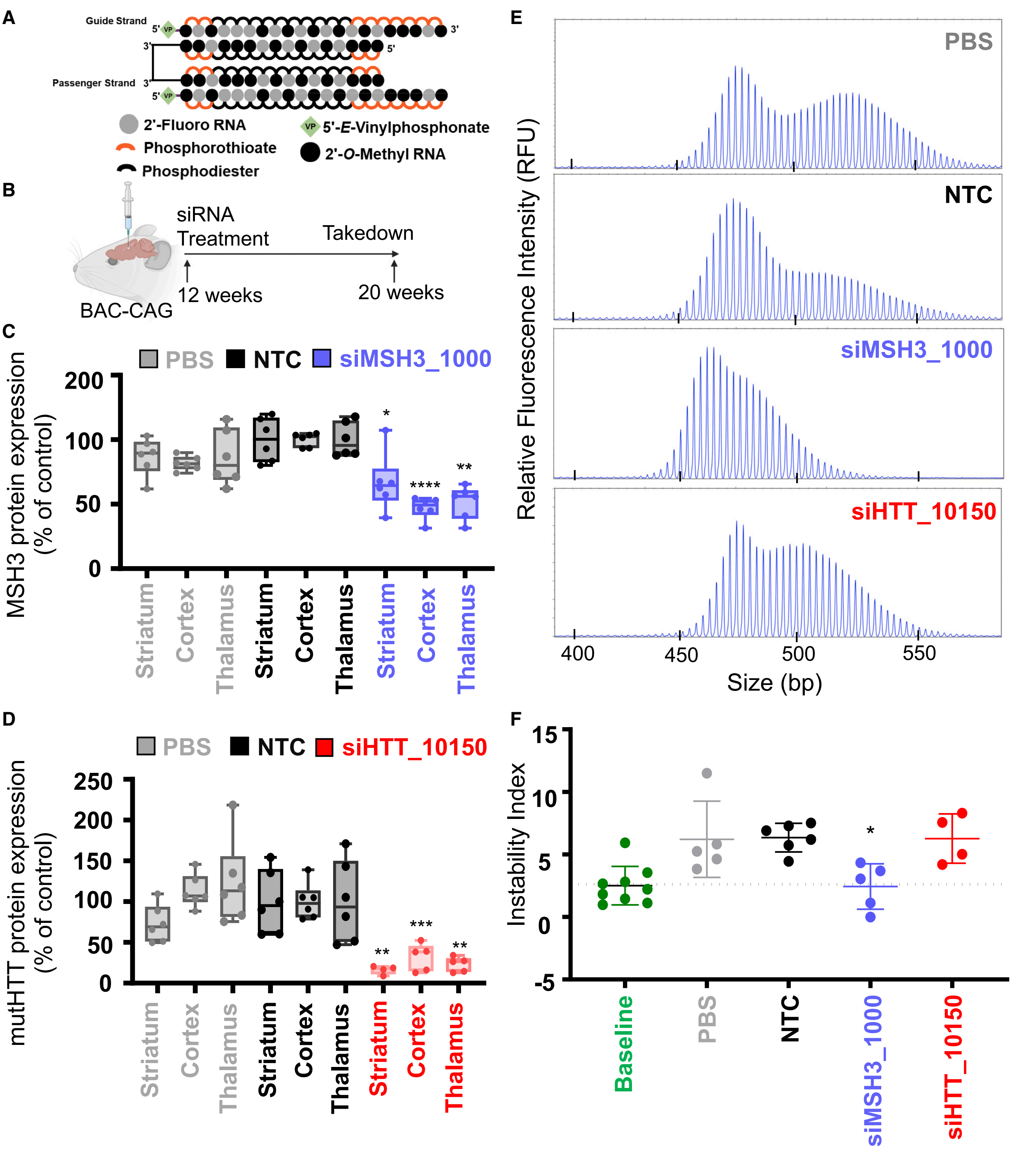

Figure 3. MSH3 silencing with di-siRNA blocks somatic repeat expansion in striatum of BAC-CAG HD mice

(A) Di-valent chemically modified siRNA structure including the chemical structure used. (B) BAC-CAG study plan, injecting groups at 12 weeks of age: PBS, NTC, siHTT_10150, and siMSH3_1000. Mice were injected with 125 μg per ventricle of di-valent siRNA and were euthanized at 20 weeks. (C) MSH3 protein measured in PBS, NTC, and siMSH3_1000 groups showing 40%–50% silencing of the Msh3 protein in the striatum, cortex, and thalamus. (D) mutHTT protein expression of PBS, NTC, and siHTT_10150 showing >90% silencing in the striatum, cortex, and thalamus. Data shown are the mean ± standard deviation. n = 5–6 mice per condition. (E) Representative

(legend continued on next page)

DISCUSSION

We demonstrate that silencing Msh3 with a single dose of di-valent siRNA blocks somatic repeat expansion for up to 4 months in mouse models of HD. Somatic expansion of CAG repeats has been identified as a critical driver of HD.[14] CAG-repeat expansion is thought to mediate its pathogenic effect through toxic downstream events at the RNA and protein level. The complexities of these downstream events have made it difficult to identify the most relevant pathogenic target for intervention.[48] Currently, direct modulation of HTT expression is the predominant therapeutic paradigm under evaluation,[49] but has shown limited clinical success.[48] Targeting the potential accelerator of pathogenesis—i.e., expansion of CAG repeats—may slow, or even stop, disease progression. Moreover, somatic repeat expansion is a key feature of other trinucleotide repeat disorders, including myotonic dystrophy and Friedreich's ataxia, making this therapeutic approach potentially applicable to any disease associated with somatic repeat expansion.

Genome-wide association study (GWAS) data have identified several MMR genes as modifiers of both HD and somatic repeat expansion, but mounting evidence suggests a strong association between lower MSH3 expression, reduced somatic repeat expansion, and slower disease onset/progression.[14,15,50] Genetic knockout of Msh3 abolishes somatic repeat expansion and thus provides independent confirmation of the critical role of Msh3 in somatic repeat expansion.[23] Reducing MSH3 is, therefore, a promising therapeutic direction for HD. To validate siRNA-mediated modulation of Msh3 *in vivo*, we chose two mouse models of HD, one with the mouse *Htt* locus and the other with the human *HTT* locus, both of which undergo CAG-repeat expansion.

We show that silencing Msh3 with the siMSH3_1000 blocks somatic repeat expansion in the Hdh^{Q111} and BAC-CAG HD models. In both models ~50% protein reduction of Msh3 blocked expansion. siMSH3_1000 was evaluated over decreasing doses; the loss of Msh3 silencing at lower doses correlated with the loss of blocking of somatic repeat expansion, suggesting that there is a threshold level of Msh3 silencing that must occur to meaningfully block somatic repeat expansion. While there is a strong relationship between the level of Msh3 silencing and somatic repeat expansion at the group level, in each of our studies, no correlation between Msh3 expression and repeat expansion was seen within individual mice. We have observed that incomplete silencing of Msh3 (~50%) blocks somatic repeat expansion. The level of Msh3 protein silencing is reduced by 4 months compared with 2 months, which suggests there is a time-dependent loss of efficacy in these compounds. In more potent sequences, such as Htt_10150, the duration can be up to 6 months.[32] Previous studies have investigated striatal somatic instability in Hdh^{Q111} mice crossed to an $Msh3^{-/-}$ or $Msh3^{+/-}$ background. These studies that found minor instability remained in Hdh^{Q111} on an $Msh3^{+/-}$ background.[23] In our Hdh^{Q111} mice, we also see that, following treatment with siMSH3_1000, there is some instability that remains. There is no statistical difference ($p > 0.05$) between the instability of untreated mice at 3 months and MSH3_1000-treated mice at 5 months. Taken together, our siRNA-mediated silencing of Msh3 does not eliminate existing instability altogether, but slows further increase in instability from the time of treatment onward. Although our datasets are consistent, it is interesting to compare genetic with siRNA-mediated silencing. Genetic knockout of one allele eliminated 50% of expression in all cells across the mouse's lifetime. Based on prior studies, target silencing with siRNA is consistent across CNS cell types.[32,51] However, levels of silencing may be more extreme early on and then reduce over time. Despite these factors, the 50% Msh3 expression following siRNA-mediated silencing is similar to the 50% Msh3 expression in the $Hdh^{111}Msh3^{+/-}$ model.

We did identify differences between siMSH3_1000 and siMSH3_1468, suggesting that the sequences of the siRNA and the target region of the *Msh3* mRNA contribute to siRNA efficacy and biological outcomes. Msh3 silencing and blockage of somatic expansion were greater with siMSH3_1000 than with siMSH3_1468, suggesting that the level of Msh3 is tightly connected to the extent of somatic expansion. Differences in Msh3 silencing between the MSH3_1000 and the MSH3_1468 sequences were clear in our 2 and 4 month study duration, where MSH3_1000 and MSH3_1468 both blocked expansion but only MSH3_1000 produced detectable protein silencing at 2 and 4 months. We expect that MSH3_1468 did silence Msh3 maximally at 1 month post-injection but declined in silencing capacity by 4 months. A duration-of-effect study will be required to measure the siRNA pharmacokinetics. In the 4 month study where mRNA was measured, we observed >50% *Msh3* mRNA silencing in both groups (70% silencing for siMSH3_1000 vs. 60% silencing for siMSH3_1468). However, at the protein level, only siMSH3_1000 was able to sustain statistically significant silencing. While the average percentage of Msh3 expression is different between groups, there is no statistical difference ($p > 0.05$) between siMSH3_1000 and siMSH3_1468 groups due to the variability between mice within treatment groups.

Across our studies, even when protein silencing was not detected at the endpoint, blockage of somatic repeat expansion was still observed. This suggests that the protein level measured at the endpoint may not reflect the Msh3 level over the study duration. The inferred level of silencing required for phenotypic outcome must be made with great care, as we measured protein silencing only at the endpoint, which may not reflect levels of silencing over the course of treatment. Further single-cell analysis would be required to link the level of Msh3 expression to somatic expansion to answer this question.

fragment analysis of the expanded CAG locus in striatum of PBS-, NTC-, siMSH3_1000-, and siHTT_10150-treated BAC-CAG mice 8 weeks post-injection. Primers are reported in the materials and methods. (F) Somatic instability index calculated with a 5% signal-to-noise threshold as described in the materials and methods. Each data point is one mouse. Instability index is compared with NTC (one-way ANOVA treatment with Dunnett's multiple comparison test; $^{*}p < 0.05$, $^{**}p < 0.01$, $^{***}p < 0.001$, $^{****}p < 0.0001$). Data shown are the mean ± standard deviation.

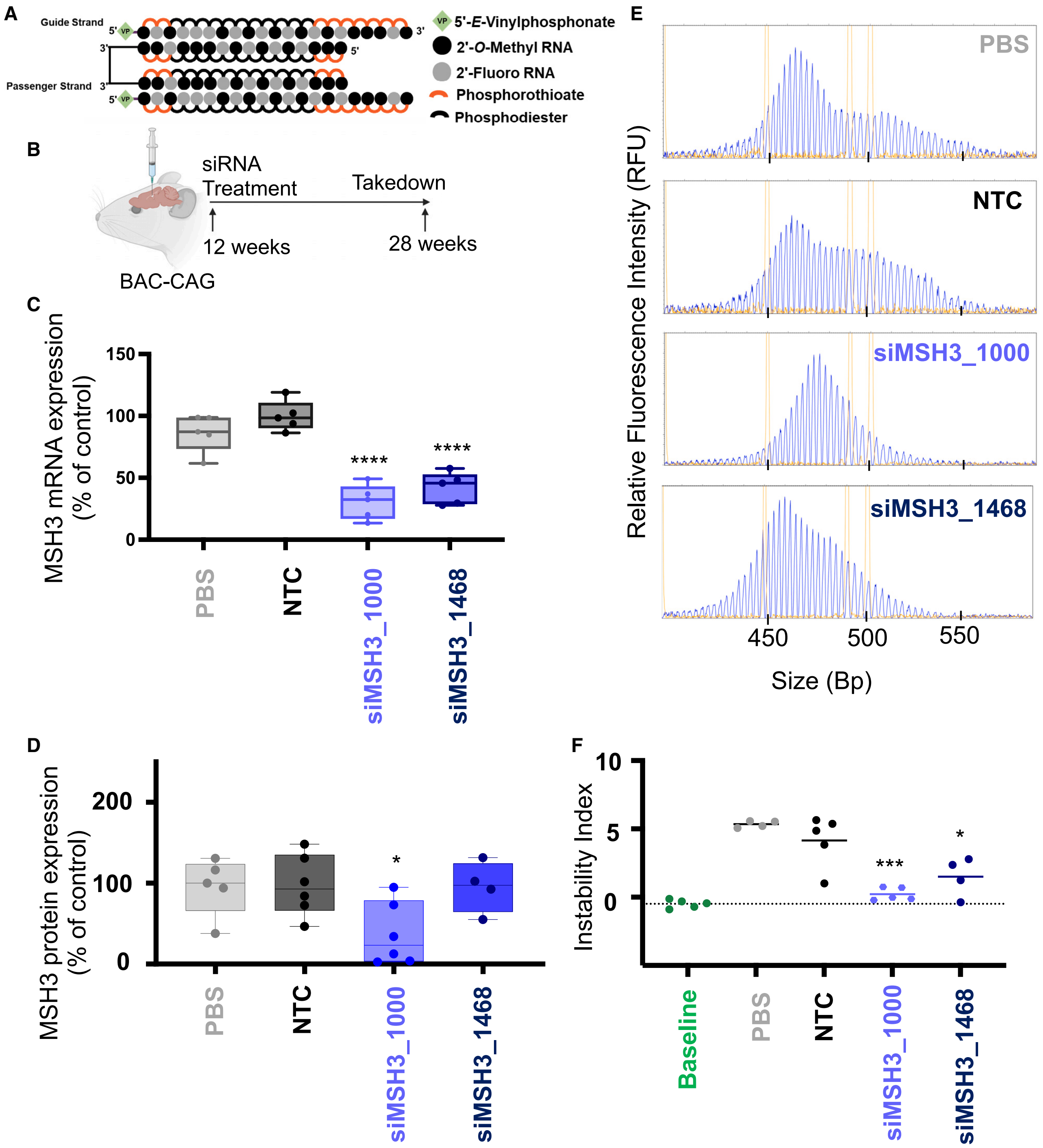

Figure 4. MSH3 silencing with di-valent MSH3_1000 and MSH3_1468 blocks somatic repeat expansion in BAC-CAG HD mice after 4 months treatment duration

(A) Di-valent chemically modified siRNA structure including the chemical structure used. (B) BAC-CAG study plan, injecting groups at 12 weeks of age: PBS, NTC, siMSH3_1000, and siMSH3_1468. Mice were injected with 125 μg per ventricle of di-valent siRNA and were euthanized at 28 weeks. (C) MSH3 mRNA measured in PBS, NTC, siMSH3_1000, and siMSH3_1468 groups in the striatum. siMSH3_1000 and siMSH3_1468 show 70% and 60% Msh3 mRNA silencing, respectively. Data shown are the mean ± standard deviation. n = 5–6 mice per condition. (D) MSH3 protein measured in PBS, NTC, siMSH3_1000 and siMSH3_1468 groups showing 60% silencing of the

(legend continued on next page)

We did notice that the extent of instability over 4 months was less robust than over 2 months in the BAC-CAG mice used. Different cohorts of BAC-CAG mice from different sources were used in these studies. We attribute the variation in instability to the CAG level and cohort (Table S2). Each study had an internal instability control (baseline and NTC groups); therefore, the conclusions about instability from each study are valid and interpretable.

We also evaluated the effect of di-siRNA silencing of HTT on somatic repeat expansion and found that it had no effect on somatic repeat expansion *in vivo*. This finding contradicts a preprint in which ASO-mediated silencing of *Htt* reduced somatic repeat expansion.[52] It is likely that the *Htt*-targeting ASOs reduce somatic repeat expansion by interfering with locus transcriptional rates by binding nascent transcripts in the nucleus.[53] By contrast, siRNA-mediated silencing of Htt mRNA in the cytoplasm would have no impact on somatic repeat expansion.

The clinical utility of targeting MSH3 in HD relies heavily on the safety of lowering its expression. MSH3 selectively recognizes long DNA loops and recruits other MMR machinery for DNA repair.[54] This highly specific role of MSH3 in MMR explains its limited association in cancers,[18,55] with no known relationship to CNS-derived tumors being reported.[21] Our findings showing no effect of Msh3 lowering on tumor-associated microsatellite instability provide further evidence for the safety of this therapeutic approach. Further, we identified no change in the expression of other MMR genes after 4 months of siRNA-mediated Msh3 silencing, which is promising. A limitation is that, while MMR genes are not changing at the mRNA level, there is a potential for MMR expression to be changing at the protein level. Ongoing research is required to study potential long-term toxicity associated with silencing of Msh3 and investigate the impact on neuronal damage markers.

The pharmacokinetics and pharmacodynamics (PK/PD) of di-siRNA make it a safe and effective drug modality for MSH3 modulation, where the majority of the injected dose is retained in the CNS. Di-valent siRNAs, when administered by CSF infusion, achieve 30%–40% retention of the injected dose in the CNS,[33] where it broadly distributes[32] to brain structures highly affected in HD and can silence a gene target for up to 6 months. Importantly, di-valent siRNAs show limited accumulation in liver and kidney and no detectable presence in the colon, a tissue in which MSH3 silencing is associated with cancer.[51,56]

While MSH3 is a clear lead target to explore for modulation of somatic repeat expansion, other genes involved in the MMR pathway might be of interest. For example, genetic knockout of MLH1 and MLH3 has been shown to have an impact on somatic repeat expansion in HD mouse models.[14,38,57,58] The inherent sequence specificity of siRNAs and their duration of effect provide a powerful therapeutic paradigm for treating neurodegenerative disorders. The discovery and development of di-valent[32] and lipophilic siRNAs[59,60] has opened up siRNA drugs to CNS indications; several compounds are now in clinical or late preclinical development.[51]

While somatic repeat expansion contributes to HD, silencing of the expanded HTT protein might still be necessary at late stages of disease.[61] Depending on disease progression at the time of treatment, simultaneous modulation of somatic repeat expansion and mutant HTT expression might be required. One advantage of siRNA is the ability to make "cocktails" of siRNA that target multiple genes simultaneously. Thus, ongoing studies are investigating the effects of combinatorial HTT and Msh3 silencing on disease progression in HD mouse models.

MATERIALS AND METHODS

Oligonucleotide synthesis, quality control, and siRNA preparation for screening

Oligonucleotides were synthesized by phosphoramidite solid-phase synthesis on a Dr Oligo 48 (Biolytic, Fremont, CA) using 2′-fluoro RNA or 2′-O-methyl RNA phosphoramidites with standard protecting groups purchased from ChemGenes (Wilmington, MA). Non-conjugated oligonucleotides were synthesized on a 500 Å UnyLinker support (ChemGenes). Cholesterol-conjugated oligonucleotides were synthesized on a 500 Å tetraethylene glycol cholesterol support (ChemGenes). Phosphoramidites were prepared at 0.1 M in anhydrous acetonitrile (ACN), except for 2′-O-methyluridine phosphoramidite, which was dissolved in anhydrous ACN containing 15% anhydrous *N*,*N*-dimethylformamide (DMF). To activate the phosphoramidites, 5-(benzylthio)-1*H*-tetrazole (BTT) (0.25 M) in anhydrous ACN was used, and the coupling time was 4 min. Capping of unreacted sites was performed using CAP A (20% 1-methyl-*1H*-imidazole in ACN) and CAP B (30% 2,6-lutidine and 20% acetic anhydride in ACN). To oxidize the phosphite (P III) center to the phosphate (P V) center, 0.05 M iodine in pyridine-water (9:1, v/v; Apex Industrial Chemicals (AIC), Aberdeen, UK) was added for 4 min. To sulfurize the phosphite centers, a 0.1 M solution of 3-[(dimethylaminomethylene)amino]-3*H*-1,2,4-dithiazole-5-thione (DDTT) in pyridine (ChemGenes) was added for 4 min. For detritylation reactions, 3% trichloroacetic acid in dichloromethane (AIC) was utilized. Post-synthesis, the columns were washed with a solution of 10% *N*,*N*-ethylethanamine (DEA) in anhydrous ACN.

For deprotection of oligonucleotides, methylamine gas (purchased from Airgas) was used for 1 h at room temperature in a gas chamber. The oligonucleotides were placed in a vacuum desiccator to help remove any remaining methylamine gas for 20 min. The columns were

Msh3 protein in the striatum with MSH3_1000 and 5% with siMSH3_1468. Data shown are the mean ± standard deviation. (E) Representative fragment analysis of the expanded CAG locus in the striatum of PBS-, NTC-, siMSH3_1000-, and siMSH3_1468-treated BAC-CAG mice 12 weeks post-injection. Primers are reported in the materials and methods. (F) Somatic instability index calculated with a 5% signal-to-noise threshold as described in the materials and methods. Each data point is one mouse. Instability index is compared with NTC (one-way ANOVA treatment with Dunnett's multiple comparison test; *p < 0.05, ***p < 0.001, ****p < 0.0001). Data shown are the mean ± standard deviation.

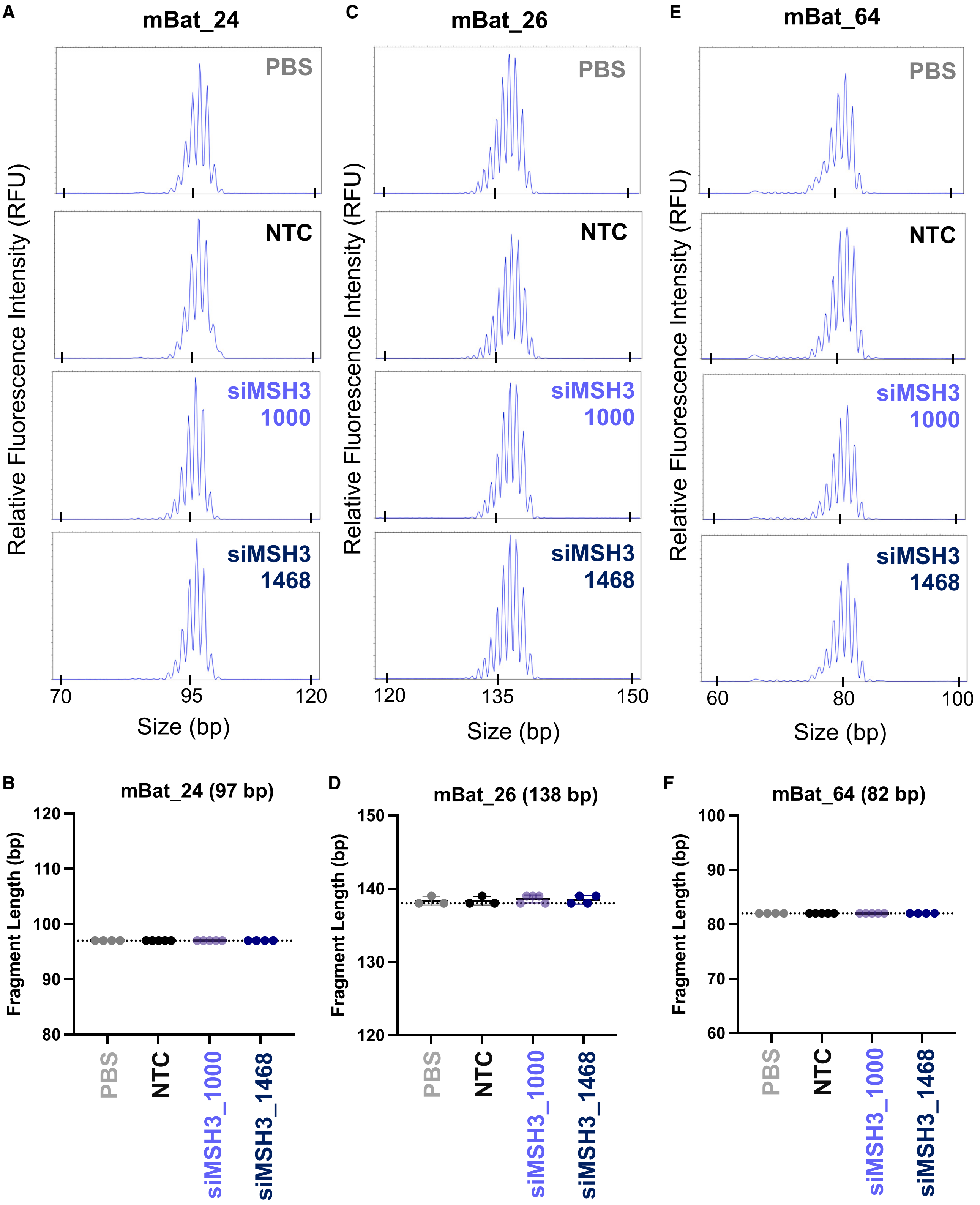

(legend on next page)

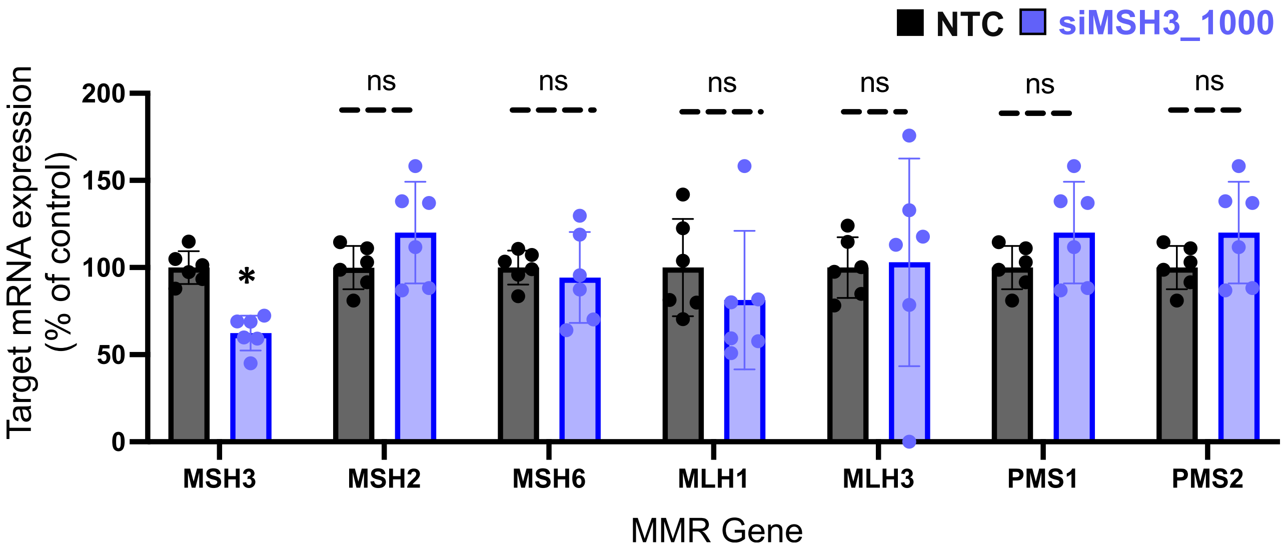

Figure 6. MSH3 silencing does not alter expression of additional mismatch repair genes

The mRNA expression of MSH3, MSH2, MSH6, MLH1, MLH3, PMS1, and PMS2 was measured by QuantiGene Singleplex assay on the medial cortex of the 4-month-treated BAC-CAG mice whose data are shown in Figure 4. Each data point is the average of three technical replicates from one mouse. Each siMSH3_1000-treated gene expression column is normalized to the NTC mouse for that gene (one-way ANOVA treatment with Dunnett's multiple comparison test, *p < 0.05). Data shown are the mean ± range.

washed with 0.1 M sodium acetate, 80% ethanol in water (five times), followed by 85% ethanol in water (five times) to precipitate oligonucleotides on the support. The ethanol was removed by heating for 5 min and placing the columns in a vacuum desiccator for 20 min. The final oligonucleotides were eluted with water. Identity of the oligonucleotides was confirmed via liquid chromatography-mass spectrometry (LC-MS).

Oligonucleotides were quantitated using a TECAN SPARK system by measuring the absorbance at 260 nm (A_{260}) of a 1:40 dilution with water. The concentrations were calculated using Beers law. The complementary antisense and sense strands were then combined to make a final siRNA concentration of 100 μM in water. Finally, this solution was heated to 95°C for 5 min and allowed to cool down to room temperature over an hour to anneal the two strands.

Synthesis of oligonucleotides for *in vivo* injections

Oligonucleotides for *in vivo* studies were synthesized on a MerMade 12 automated oligonucleotide synthesizer. Di-valent oligonucleotides were synthesized on a solid support that has been previously published.[32] For oligonucleotides that required 5′-(*E*)-vinyl tetraphosphonate (pivaloyloxymethyl), 2′-*O*-methyl-uridine 3′-CE phosphoramidite (VP) was purchased from Hongene. Di-valent oligonucleotides were synthesized on a modified solid support.[32] The synthesis cycle procedure is the same as previously discussed for screening. For deprotection of VP-containing oligonucleotides, 3% DEA in ammonia hydroxide was used and heated at 30°C for 20 h. Di-valent oligonucleotides were deprotected using an aqueous ammonia hydroxide (28%–30% in water):methylamine solution (40% in water) (1:1, v/v) at room temperature for 2 h. The solutions were evaporated to dryness. Crude material was dissolved in water and filtered to remove controlled pore glass. The crude material was purified on an Agilent 1200 Prep HPLC system using ion exchange. After purification, oligonucleotides were desalted on an AKTA FPLC with Sephadex columns. Quantitation of oligonucleotides was performed on a Nanodrop system. To anneal siRNA, the antisense and sense strands were added together and heated to 95°C for 5 min before being allowed to cool to room temperature slowly.

Cell culture

HeLa (#CCL-2), Neuro-2a (#CCL-131), and LLC-MK2 (#CCL-7) cells were purchased from the American Type Culture Collection (ATCC). HeLa and LLC-MK2 cells were maintained in Dulbecco's modified Eagle's medium (DMEM) (Cellgro, #10-013CV) with 10% fetal bovine serum (FBS) (Gibco, #26140), and Neuro-2a cells were maintained in EMEM (ATCC, #30-2003) with 10% FBS. Both cell lines were cultured at 37°C and 5% CO_2. Cells were split once confluent and were limited to 20 passages.

Figure 5. MSH3 silencing with di-valent siMSH3_1000 or si-MSH3_1468 does not affect microsatellite instability

Microsatellite instability (MSI) traces of the (A) mBAT 24, (C) mBAT 26, or (E) mBAT 64 mononucleotide repeat locus. MSI was compared between PBS, NTC, siMSH3_1000, and siMSH3_1468. MSI quantitation at the (B) mBAT 24, (D) mBAT 26, or (F) mBAT 64 loci. Traces were analyzed with Thermo Fisher Cloud PeakScanner. Primers are reported in the materials and methods. MSI is compared with NTC (one-way ANOVA). n = 4–6 mice per condition. Data shown are the mean ± standard deviation.

Screening and dose responses

For screening, siRNA was diluted in OptiMeM (Carlsbad, CA; 31985-088) to double their final concentration, then 50 μL of this dilution was placed in triplicate on a 96 well plate. Cells were trypsinized, centrifuged, and resuspended in 6% medium. This suspension was counted and diluted in 6% FBS medium so that there were 8,000–12,000 cells per 50 μL. These cells were added to the siRNA in the 96 well plate. These additions resulted in a final FBS amount of 3% and 1.5 μM concentration of siRNA. Plates were incubated for 72 h at 5% CO_2 and 37°C.

For 7-point dose-response studies, siRNA was diluted in OptiMeM and then serially diluted (2× dilution) and plated in triplicate on 96 well plates. For the addition of cells, the same procedure outlined for screening was followed.

Animal experiments

All animal care was in accordance with institutional guidelines. Animal experiments were approved by the UMass Chan Medical School IACUC (protocol 202000010). Hdh^{Q111} mice were provided by The Jackson Laboratory (Bar Harbor, ME, USA) at 6–8 weeks of age. Hdh^{Q111} mice are The Jackson Laboratory strain 003456 on the C57BL6 background. BAC-CAG mice are The Jackson Laboratory strain 037050 on the FVB background. All strain and mouse CAG-repeat information can be found in Table S2. At 12 weeks of age, the mice were bilaterally i.c.v. injected with 10 μL of siRNA (5 μL per ventricle) in PBS, at a rate of 500 nL min^{-1}. The coordinates from bregma were −0.2 mm AP, ±1.0 mm mediolateral, and −2.5 mm dorsoventral. Mice were anesthetized throughout the procedure using 1.2% Avertin or isoflurane. After 2 months, the mice were euthanized. Mice were perfused with PBS, and half brains were frozen in OCT for RNAScope. For protein analysis, 1.5 × 1.5 mm punches were flash frozen; for mRNA analysis, punches were placed in RNAlater for 24 h at 4°C.

Western blot analysis

Frozen tissue punches from striatum, medial cortex, posterior cortex, and thalamus were homogenized on ice in 75 μL 10 mM HEPES (pH 7.2), 250 mM sucrose, 1 mM EDTA + protease inhibitor tablet (Roche; complete, EDTA-free) + 1 mM NaF +1 mM Na_3VO_4, and sonicated for 10 s. Protein concentration was determined using the Bradford method (Bio-Rad). Equal amounts of protein (10 μg) were separated by SDS-PAGE and analyzed by western blot using antibodies to huntingtin (1:2,000, Ab1, aa1-17),[62] MSH3 (1:500, Santa Cruz), β-tubulin (1:5,000, Sigma), and GAPDH (1:10,000, Millipore) as previously described.[63] Bands were visualized with SuperSignal West Pico PLUS chemiluminescence substrate (Pierce) and images were obtained with a CCD camera (AlphaInnotech). Pixel intensity quantification was performed using ImageJ software (NIH) by manually circling each band and multiplying the area by the average intensity to obtain the total intensity for each band and normalizing the signal to the tubulin or GAPDH loading control.

Fragment analysis

Genomic DNA was extracted from ∼10 mg punches from selected brain regions using the solid tissue protocol from the IBI gMax Mini Kit (IBI cat. no. IB47218). DNA concentrations were determined using the Qubit Flex fluorometer. The CAG-repeat region of the Htt gene was amplified in 80 μL PCRs using forward primer CAG1 (5′-[6FAM]-ATGAAGGCCTTCGAGTCCCTCAAGTCCTTC-3′) and reverse primer Hu3 (5′-GGCGGCTGAGGAAGCTGAGGA-3′). Each 80 μL PCR consisted of 8 μL AmpliTaq buffer, 8 μL DMSO, 4 μL BSA (20 mg/mL), 8 μL GC enhancer, 3.2 μL 25 mM $MgCl_2$, 8 μL 2 mM dNTPs, 6.4 μL 10 μM forward primer, 6.4 μL 10 mM reverse primer, 19.2 μL H_2O, and 0.8 μL AmpliTaq 360 Taq polymerase. Thermocycling conditions were as follows: An initial denaturation of 1 min 30 s at 94°C; then 35 cycles of 30 s at 94°C, 30 s at 63.5°C, and 1 min 30 s at 72°C; followed by a final annealing step of 10 min at 72°C. The 80 μL PCR product was concentrated down to 20 μL using the GeneJET PCR purification kit (Thermo Fisher, cat. no. K0702). PCR products were eluted into 20 μL of water. Concentrated PCR products were sent to GeneWiz for fragment analysis. Traces were visualized using Peak Scanner 2 software, and expansion indices were calculated using a custom R script based on somatic instability index calculations.[44]

Statistics

When comparing two or more groups, one-way ANOVA vs. control (NTC, PBS) with Dunnett's *post hoc* analysis was used. When comparing two groups, Student's t test was used; $^{*}p \leq 0.05$, $^{**}p \leq 0.01$, $^{***}p \leq 0.001$, and $^{****}p \leq 0.000$. For western blot, QuantiGene, and fragment analysis quantitation, technical replicates were performed.

DATA AVAILABILITY

All data presented in the main text or the supplemental information are available from the corresponding author upon request.

SUPPLEMENTAL INFORMATION

Supplemental information can be found online at https://doi.org/10.1016/j.ymthe.2023.05.006.

ACKNOWLEDGMENTS

The authors acknowledge all Khvorova and Aronin lab members who kindly reviewed the manuscript and provided editorial suggestions. The authors thank Lori Kennington, Vicky Benoit, and Rachael Miller for assistance in breeding and genotyping mice. M.D. and N.A. are funded under NIH U01 NS114098. M.D. thanks CHDI-6367 and the Dake Family Fund as funding sources. N.A. is funded under NIH R01 NS106245. N.A. and A.K. thank the CHDI Foundation. A.K. is funded under NIH R35 GM131839 and S10 OD020012.

AUTHOR CONTRIBUTIONS

Conceptualization was performed by C.F., A.K., J. Buchwald, D.O., and J. Belgrad. Visualization was performed by D.O., J. Buchwald, A.K., N.A., and M.D. Methodology was designed by D.O., J. Buchwald, C.F., and A.K. Investigation was performed by D.O., J. Buchwald, A.K., C.F., A.S., E.S., C.M., E.M., J.B., S.L., D.M., Z.K., V.H., and K.M. Funding acquisition was done by A.K., N.A., M.D., and J.C. Project administration and supervision were performed by A.K., N.A., M.D., and J.C. Writing the original draft was completed

by D.O., J. Buchwald, and A.K. Reviewing and editing the original draft was completed by D.O., J. Buchwald, A.K., N.A., and M.D.

DECLARATION OF INTERESTS

A.K. and N.A. are co-founders of Atalanta. A.K. and N.A. are on the scientific advisory board of Atalanta. N.A. is on the scientific advisory board of Huntington's Disease Society for America and that of Biogen and has licensing to Spark Therapeutics. A.K. has a financial interest in Atalanta. The authors have patents on MSH3 as a therapeutic target and the methodology described in the paper.

REFERENCES

1. Tabrizi, S.J., Flower, M.D., Ross, C.A., and Wild, E.J. (2020). Huntington disease: new insights into molecular pathogenesis and therapeutic opportunities. Nat. Rev. Neurol. *16*, 529–546. https://doi.org/10.1038/s41582-020-0389-4.
2. Wild, E.J., and Tabrizi, S.J. (2017). Therapies targeting DNA and RNA in Huntington's disease. Lancet Neurol. *16*, 837–847. https://doi.org/10.1016/S1474-4422(17)30280-6.
3. Tabrizi, S.J., Estevez-Fraga, C., van Roon-Mom, W.M.C., Flower, M.D., Scahill, R.I., Wild, E.J., Muñoz-Sanjuan, I., Sampaio, C., Rosser, A.E., and Leavitt, B.R. (2022). Potential disease-modifying therapies for Huntington's disease: lessons learned and future opportunities. Lancet Neurol. *21*, 645–658. https://doi.org/10.1016/S1474-4422(22)00121-1.
4. Ghosh, R., and Tabrizi, S.J. (2018). Clinical features of huntington's disease. In Polyglutamine Disorders, C. Nóbrega and L. Pereira de Almeida, eds. (Springer International Publishing), pp. 1–28. https://doi.org/10.1007/978-3-319-71779-1_1.
5. McColgan, P., and Tabrizi, S.J. (2018). Huntington's disease: a clinical review. Eur. J. Neurol. *25*, 24–34. https://doi.org/10.1111/ene.13413.
6. Tabrizi, S.J., Schobel, S., Gantman, E.C., Mansbach, A., Borowsky, B., Konstantinova, P., Mestre, T.A., Panagoulias, J., Ross, C.A., Zauderer, M., et al. (2022). A biological classification of Huntington's disease: the Integrated Staging System. Lancet Neurol. *21*, 632–644. https://doi.org/10.1016/S1474-4422(22)00120-X.
7. Monckton, D.G. (2021). The contribution of somatic expansion of the CAG repeat to symptomatic development in huntington's disease: a historical perspective. J. Huntingtons Dis. *10*, 7–33. https://doi.org/10.3233/JHD-200429.
8. Swami, M., Hendricks, A.E., Gillis, T., Massood, T., Mysore, J., Myers, R.H., and Wheeler, V.C. (2009). Somatic expansion of the Huntington's disease CAG repeat in the brain is associated with an earlier age of disease onset. Hum. Mol. Genet. *18*, 3039–3047. https://doi.org/10.1093/hmg/ddp242.
9. Nouspikel, T., and Hanawalt, P.C. (2000). Terminally differentiated human neurons repair transcribed genes but display attenuated global DNA repair and modulation of repair gene expression. Mol. Cell. Biol. *20*, 1562–1570. https://doi.org/10.1128/mcb.20.5.1562-1570.2000.
10. Aronin, N., Chase, K., Young, C., Sapp, E., Schwarz, C., Matta, N., Kornreich, R., Landwehrmeyer, B., Bird, E., Beal, M.F., et al. (1995). CAG expansion affects the expression of mutant Huntingtin in the Huntington's disease brain. Neuron *15*, 1193–1201. https://doi.org/10.1016/0896-6273(95)90106-x.
11. Hu, T., Morten, M.J., and Magennis, S.W. (2021). Conformational and migrational dynamics of slipped-strand DNA three-way junctions containing trinucleotide repeats. Nat. Commun. *12*, 204. https://doi.org/10.1038/s41467-020-20426-3.
12. Khomyakova, E.B., Petrova, M.V., Minyat, E.E., and Ivanov, V.I. (1998). Slipped loop structure of DNA: a specific nucleotide sequence forms only one unique conformer. FEBS Lett. *422*, 265–268. https://doi.org/10.1016/S0014-5793(98)00022-2.
13. Axford, M.M., Wang, Y.-H., Nakamori, M., Zannis-Hadjopoulos, M., Thornton, C.A., and Pearson, C.E. (2013). Detection of slipped-DNAs at the trinucleotide repeats of the myotonic dystrophy type I disease locus in patient tissues. Plos Genet. *9*, e1003866. https://doi.org/10.1371/journal.pgen.1003866.
14. Lee, J.-M., Correia, K., Loupe, J., Kim, K.-H., Barker, D., Hong, E.P., Chao, M.J., Long, J.D., Lucente, D., Vonsattel, J.P.G., et al. (2019). CAG repeat not polyglutamine length determines timing of huntington's disease onset. Cell *178*, 887–900.e14. https://doi.org/10.1016/j.cell.2019.06.036.
15. Flower, M., Lomeikaite, V., Ciosi, M., Cumming, S., Morales, F., Lo, K., Hensman Moss, D., Jones, L., Holmans, P., TRACK-HD Investigators, et al. (2019). MSH3 modifies somatic instability and disease severity in Huntington's and myotonic dystrophy type 1. Brain. *142*, 1876–1886. https://doi.org/10.1093/brain/awz115.
16. Moss, D.J.H., Pardiñas, A.F., Langbehn, D., Lo, K., Leavitt, B.R., Roos, R., Durr, A., Mead, S., TRACK-HD investigators, REGISTRY investigators, et al. (2017). Identification of genetic variants associated with Huntington's disease progression: a genome-wide association study. Lancet Neurol. *16*, 701–711. https://doi.org/10.1016/s1474-4422(17)30161-8.
17. Gupta, D., and Heinen, C.D. (2019). The mismatch repair-dependent DNA damage response: mechanisms and implications. DNA Repair *78*, 60–69. https://doi.org/10.1016/j.dnarep.2019.03.009.
18. Pećina-Šlaus, N., Kafka, A., Salamon, I., and Bukovac, A. (2020). Mismatch repair pathway, genome stability and cancer. Front. Mol. Biosci. *7*, 122. https://doi.org/10.3389/fmolb.2020.00122.
19. Adam, R., Spier, I., Zhao, B., Kloth, M., Marquez, J., Hinrichsen, I., Kirfel, J., Tafazzoli, A., Horpaopan, S., Uhlhaas, S., et al. (2016). Exome sequencing identifies biallelic MSH3 germline Mutations as a recessive subtype of colorectal adenomatous polyposis. Am. J. Hum. Genet. *99*, 337–351. https://doi.org/10.1016/j.ajhg.2016.06.015.
20. Young, S.J., and West, S.C. (2021). Coordinated roles of SLX4 and MutSβ in DNA repair and the maintenance of genome stability. Crit. Rev. Biochem. Mol. Biol. *56*, 157–177. https://doi.org/10.1080/10409238.2021.1881433.
21. Zhao, M., Liu, Y., Ding, G., Qu, D., and Qu, H. (2021). Online database for brain cancer-implicated genes: exploring the subtype-specific mechanisms of brain cancer. BMC Genomics *22*, 458. https://doi.org/10.1186/s12864-021-07793-x.
22. Stone, J., Donaldson, J., Powell, S., Allen, N., Massey, T., and Jones, L. (2021). C01 the effect of mismatch repair proteins in a huntington's disease cellular model. J. Neurol. Neurosurg. Psychiatry *92*, A10. https://doi.org/10.1136/jnnp-2021-EHDN.25.
23. Dragileva, E., Hendricks, A., Teed, A., Gillis, T., Lopez, E.T., Friedberg, E.C., Kucherlapati, R., Edelmann, W., Lunetta, K.L., MacDonald, M.E., and Wheeler, V.C. (2009). Intergenerational and striatal CAG repeat instability in Huntington's disease knock-in mice involve different DNA repair genes. Neurobiol. Dis. *33*, 37–47. https://doi.org/10.1016/j.nbd.2008.09.014.
24. Zhang, M.M., Bahal, R., Rasmussen, T.P., Manautou, J.E., and Zhong, X.-b. (2021). The growth of siRNA-based therapeutics: updated clinical studies. Biochem. Pharmacol. *189*, 114432. https://doi.org/10.1016/j.bcp.2021.114432.
25. Hu, B., Zhong, L., Weng, Y., Peng, L., Huang, Y., Zhao, Y., and Liang, X.-J. (2020). Therapeutic siRNA: state of the art. Signal Transduct. Target. Ther. *5*, 101. https://doi.org/10.1038/s41392-020-0207-x.
26. Hassler, M.R., Turanov, A.A., Alterman, J.F., Haraszti, R.A., Coles, A.H., Osborn, M.F., Echeverria, D., Nikan, M., Salomon, W.E., Roux, L., et al. (2018). Comparison of partially and fully chemically-modified siRNA in conjugate-mediated delivery in vivo. Nucleic Acids Res. *46*, 2185–2196. https://doi.org/10.1093/nar/gky037.
27. McKenzie, L.K., El-Khoury, R., Thorpe, J.D., Damha, M.J., and Hollenstein, M. (2021). Recent progress in non-native nucleic acid modifications. Chem. Soc. Rev. *50*, 5126–5164. https://doi.org/10.1039/D0CS01430C.
28. Deleavey, G.F., and Damha, M.J. (2012). Designing chemically modified oligonucleotides for targeted gene silencing. Chem. Biol. *19*, 937–954. https://doi.org/10.1016/j.chembiol.2012.07.011.
29. Khvorova, A., and Watts, J.K. (2017). The chemical evolution of oligonucleotide therapies of clinical utility. Nat. Biotechnol. *35*, 238–248. https://doi.org/10.1038/nbt.3765.
30. Setten, R.L., Rossi, J.J., and Han, S.-p. (2019). The current state and future directions of RNAi-based therapeutics. Nat. Rev. Drug Discov. *18*, 421–446. https://doi.org/10.1038/s41573-019-0017-4.
31. Ly, S., Echeverria, D., Sousa, J., and Khvorova, A. (2020). Single-stranded phosphorothioated regions enhance cellular uptake of cholesterol-conjugated siRNA but not silencing efficacy. Mol. Ther. Nucleic Acids *21*, 991–1005. https://doi.org/10.1016/j.omtn.2020.07.029.
32. Alterman, J.F., Godinho, B.M., Hassler, M.R., Ferguson, C.M., Echeverria, D., Sapp, E., Haraszti, R.A., Coles, A.H., Conroy, F., Miller, R., et al. (2019). A divalent siRNA chemical scaffold for potent and sustained modulation of gene expression

throughout the central nervous system. Nat. Biotechnol. *37*, 884–894. https://doi.org/10.1038/s41587-019-0205-0.

33. Ferguson, C.M., Godinho, B.M., Alterman, J.F., Coles, A.H., Hassler, M., Echeverria, D., Gilbert, J.W., Knox, E.G., Caiazzi, J., Haraszti, R.A., et al. (2021). Comparative route of administration studies using therapeutic siRNAs show widespread gene modulation in Dorset sheep. JCI Insight *6*, e152203. https://doi.org/10.1172/jci.insight.152203.

34. Shmushkovich, T., Monopoli, K.R., Homsy, D., Leyfer, D., Betancur-Boissel, M., Khvorova, A., and Wolfson, A.D. (2018). Functional features defining the efficacy of cholesterol-conjugated, self-deliverable, chemically modified siRNAs. Nucleic Acids Res. *46*, 10905–10916. https://doi.org/10.1093/nar/gky745.

35. Haraszti, R.A., Roux, L., Coles, A.H., Turanov, A.A., Alterman, J.F., Echeverria, D., Godinho, B.M., Aronin, N., and Khvorova, A. (2017). 5´-Vinylphosphonate improves tissue accumulation and efficacy of conjugated siRNAs in vivo. Nucleic Acids Res. *45*, 7581–7592. https://doi.org/10.1093/nar/gkx507.

36. Tang, Q., Sousa, J., Echeverria, D., Fan, X., Hsueh, Y.-C., Afshari, K., MeHugh, N., Cooper, D.A., Vangjeli, L., Monopoli, K., et al. (2022). RNAi-based modulation of IFN-γ signaling in skin. Mol. Ther. *30*, 2709–2721. https://doi.org/10.1016/j.ymthe.2022.04.019.

37. Ferguson, C.M., Echeverria, D., Hassler, M., Ly, S., and Khvorova, A. (2020). Cell type impacts accessibility of mRNA to silencing by RNA interference. Mol. Ther. Nucleic Acids *21*, 384–393. https://doi.org/10.1016/j.omtn.2020.06.006.

38. Roy, J.C.L., Vitalo, A., Andrew, M.A., Mota-Silva, E., Kovalenko, M., Burch, Z., Nhu, A.M., Cohen, P.E., Grabczyk, E., Wheeler, V.C., and Mouro Pinto, R. (2021). Somatic CAG expansion in Huntington's disease is dependent on the MLH3 endonuclease domain, which can be excluded via splice redirection. Nucleic Acids Res. *49*, 3907–3918. https://doi.org/10.1093/nar/gkab152.

39. Trettel, F., Rigamonti, D., Hilditch-Maguire, P., Wheeler, V.C., Sharp, A.H., Persichetti, F., Cattaneo, E., and MacDonald, M.E. (2000). Dominant phenotypes produced by the HD mutation in STHdhQ111 striatal cells. Hum. Mol. Genet. *9*, 2799–2809. https://doi.org/10.1093/hmg/9.19.2799.

40. Lee, J.-M., Pinto, R.M., Gillis, T., St Claire, J.C., and Wheeler, V.C. (2011). Quantification of age-dependent somatic CAG repeat instability in Hdh CAG knock-in mice reveals different expansion dynamics in striatum and liver. PLoS One *6*, e23647. https://doi.org/10.1371/journal.pone.0023647.

41. Wheeler, V.C., Auerbach, W., White, J.K., Srinidhi, J., Auerbach, A., Ryan, A., Duyao, M.P., Vrbanac, V., Weaver, M., Gusella, J.F., et al. (1999). Length-dependent gametic CAG repeat instability in the huntington's disease knock-in mouse. Hum. Mol. Genet. *8*, 115–122. https://doi.org/10.1093/hmg/8.1.115.

42. Parmar, R.G., Brown, C.R., Matsuda, S., Willoughby, J.L.S., Theile, C.S., Charissé, K., Foster, D.J., Zlatev, I., Jadhav, V., Maier, M.A., et al. (2018). Facile synthesis, geometry, and 2′-substituent-dependent in vivo activity of 5′-(E)- and 5′-(Z)-Vinylphosphonate-Modified siRNA conjugates. J. Med. Chem. *61*, 734–744. https://doi.org/10.1021/acs.jmedchem.7b01147.

43. Alterman, J.F., Hall, L.M., Coles, A.H., Hassler, M.R., Didiot, M.-C., Chase, K., Abraham, J., Sottosanti, E., Johnson, E., Sapp, E., et al. (2015). Hydrophobically modified siRNAs silence huntingtin mRNA in primary neurons and mouse brain. Mol. Ther. Nucleic Acids *4*, e266. https://doi.org/10.1038/mtna.2015.38.

44. Lee, J.-M., Zhang, J., Su, A.I., Walker, J.R., Wiltshire, T., Kang, K., Dragileva, E., Gillis, T., Lopez, E.T., Boily, M.-J., et al. (2010). A novel approach to investigate tissue-specific trinucleotide repeat instability. BMC Syst. Biol. *4*, 29. https://doi.org/10.1186/1752-0509-4-29.

45. Gu, X., Richman, J., Langfelder, P., Wang, N., Zhang, S., Bañez-Coronel, M., Wang, H.-B., Yang, L., Ramanathan, L., Deng, L., et al. (2022). Uninterrupted CAG repeat drives striatum-selective transcriptionopathy and nuclear pathogenesis in human Huntingtin BAC mice. Neuron *110*, 1173–1192.e7. https://doi.org/10.1016/j.neuron.2022.01.006.

46. Bonneville, R., Krook, M.A., Kautto, E.A., Miya, J., Wing, M.R., Chen, H.-Z., Reeser, J.W., Yu, L., and Roychowdhury, S. (2017). Landscape of microsatellite instability across 39 cancer types. JCO Precis. Oncol. *2017*, 1–15. https://doi.org/10.1200/PO.17.00073.

47. Bacher, J.W., Abdel Megid, W.M., Kent-First, M.G., and Halberg, R.B. (2005). Use of mononucleotide repeat markers for detection of microsatellite instability in mouse tumors. Mol. Carcinog. *44*, 285–292. https://doi.org/10.1002/mc.20146.

48. Kwon, D. (2021). Failure of genetic therapies for Huntington's devastates community. Nature *593*, 180. https://doi.org/10.1038/d41586-021-01177-7.

49. Tabrizi, S.J., Ghosh, R., and Leavitt, B.R. (2019). Huntingtin lowering strategies for disease modification in huntington's disease. Neuron *101*, 801–819. https://doi.org/10.1016/j.neuron.2019.01.039.

50. Goold, R., Hamilton, J., Menneteau, T., Flower, M., Bunting, E.L., Aldous, S.G., Porro, A., Vicente, J.R., Allen, N.D., Wilkinson, H., et al. (2021). FAN1 controls mismatch repair complex assembly via MLH1 retention to stabilize CAG repeat expansion in Huntington's disease. Cell Rep. *36*, 109649. https://doi.org/10.1016/j.celrep.2021.109649.

51. Ferguson, C., Hildebrand, S., Godinho, B.M., Buchwald, J., Echeverria, D., Coles, A., Grigorenko, A., Vanjielli, L., Sousa, J., McHugh, N., et al. (2022). Silencing of ApoE with divalent siRNAs drives activation of immune clearance pathways and improves amyloid pathology in mouse models of Alzheimer's Disease. Preprint at bioRxiv. https://doi.org/10.1101/2022.06.28.498012.

52. Coffey, S.R., Andrew, M., Ging, H., Hamilton, J., Flower, M., Kovalenko, M., Bragg, R.M., Cantle, J.P., McHugh, C.A., Carrillo, J.M., et al. (2020). Huntingtin lowering reduces somatic instability at CAG-expanded loci. Preprint at bioRxiv. https://doi.org/10.1101/2020.07.23.218347.

53. Lai, F., Damle, S.S., Ling, K.K., and Rigo, F. (2020). Directed RNase H cleavage of nascent transcripts causes transcription termination. Mol. Cell *77*, 1032–1043.e4. https://doi.org/10.1016/j.molcel.2019.12.029.

54. Tseng-Rogenski, S.S., Munakata, K., Choi, D.Y., Martin, P.K., Mehta, S., Koi, M., Zheng, W., Zhang, Y., and Carethers, J.M. (2020). The human DNA mismatch repair protein MSH3 contains nuclear localization and export signals that enable nuclear-cytosolic shuttling in response to inflammation. Mol. Cell. Biol. *40*, e00029-20–e00020. https://doi.org/10.1128/MCB.00029-20.

55. Martin, S.A., Lord, C.J., and Ashworth, A. (2010). Therapeutic targeting of the DNA mismatch repair pathway. Clin. Cancer Res. *16*, 5107–5113. https://doi.org/10.1158/1078-0432.Ccr-10-0821.

56. Orimo, H., Nakajima, E., Yamamoto, M., Ikejima, M., Emi, M., and Shimada, T. (2000). Association between single nucleotide polymorphisms in the hMSH3 gene and sporadic colon cancer with microsatellite instability. J. Hum. Genet. *45*, 228–230. https://doi.org/10.1007/s100380070031.

57. Pinto, R.M., Dragileva, E., Kirby, A., Lloret, A., Lopez, E., St Claire, J., Panigrahi, G.B., Hou, C., Holloway, K., Gillis, T., et al. (2013). Mismatch repair genes Mlh1 and Mlh3 modify CAG instability in huntington's disease mice: genome-wide and candidate approaches. Plos Genet. *9*, e1003930. https://doi.org/10.1371/journal.pgen.1003930.

58. Lee, J.-M., Chao, M.J., Harold, D., Abu Elneel, K., Gillis, T., Holmans, P., Jones, L., Orth, M., Myers, R.H., Kwak, S., et al. (2017). A modifier of Huntington's disease onset at the MLH1 locus. Hum. Mol. Genet. *26*, 3859–3867. https://doi.org/10.1093/hmg/ddx286.

59. Osborn, M.F., and Khvorova, A. (2018). Improving siRNA delivery in vivo through lipid conjugation. Nucleic Acid Ther. *28*, 128–136. https://doi.org/10.1089/nat.2018.0725.

60. Brown, K.M., Nair, J.K., Janas, M.M., Anglero-Rodriguez, Y.I., Dang, L.T.H., Peng, H., Theile, C.S., Castellanos-Rizaldos, E., Brown, C., Foster, D., et al. (2022). Expanding RNAi therapeutics to extrahepatic tissues with lipophilic conjugates. Nat. Biotechnol. *40*, 1500–1508. https://doi.org/10.1038/s41587-022-01334-x.

61. Genetic Modifiers of Huntington's Disease GeM-HD Consortium Electronic address gusella@helixmghharvardedu, Genetic Modifiers of Huntington's Disease GeM-HD Consortium (2019). CAG repeat not polyglutamine length determines timing of huntington's disease onset. Cell *178*, 887–900.e14. https://doi.org/10.1016/j.cell.2019.06.036.

62. DiFiglia, M., Sapp, E., Chase, K., Schwarz, C., Meloni, A., Young, C., Martin, E., Vonsattel, J.-P., Carraway, R., Reeves, S.A., et al. (1995). Huntingtin is a cytoplasmic protein associated with vesicles in human and rat brain neurons. Neuron *14*, 1075–1081. https://doi.org/10.1016/0896-6273(95)90346-1.

63. Sapp, E., Seeley, C., Iuliano, M., Weisman, E., Vodicka, P., DiFiglia, M., and Kegel-Gleason, K.B. (2020). Protein changes in synaptosomes of Huntington's disease knock-in mice are dependent on age and brain region. Neurobiol. Dis. *141*, 104950. https://doi.org/10.1016/j.nbd.2020.104950.

YMTHE, Volume *31*

Supplemental Information

Di-valent siRNA-mediated silencing of MSH3 blocks somatic repeat expansion in mouse models of Huntington's disease

Daniel O'Reilly, Jillian Belgrad, Chantal Ferguson, Ashley Summers, Ellen Sapp, Cassandra McHugh, Ella Mathews, Adel Boudi, Julianna Buchwald, Socheata Ly, Dimas Moreno, Raymond Furgal, Eric Luu, Zachary Kennedy, Vignesh Hariharan, Kathryn Monopoli, X. William Yang, Jeffery Carroll, Marian DiFiglia, Neil Aronin, and Anastasia Khvorova

SUPPLEMENTAL MATERIALS

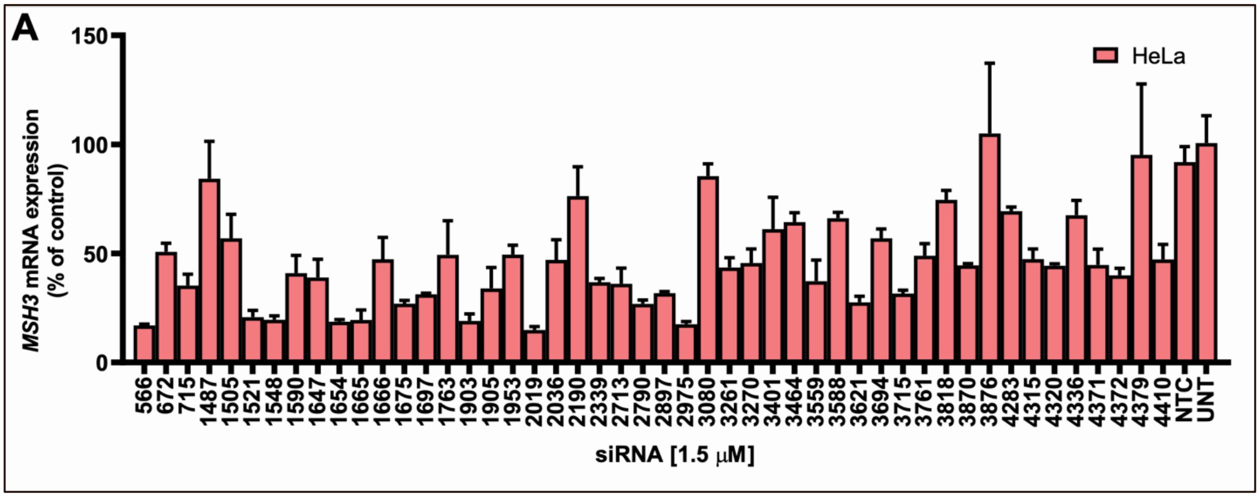

Figure S1: Identification of potent siRNA targeting *Msh3*. *In vitro* screen results for various MSH3-targeting sequences, aligned by 5'-3' location across the target gene. HeLa cells treated 1.5 μM siRNA for 72 hours. mRNA quantified with Quantigene assay and *Msh3* expression represented as abundance normalized to HPRT and relative to NTC.

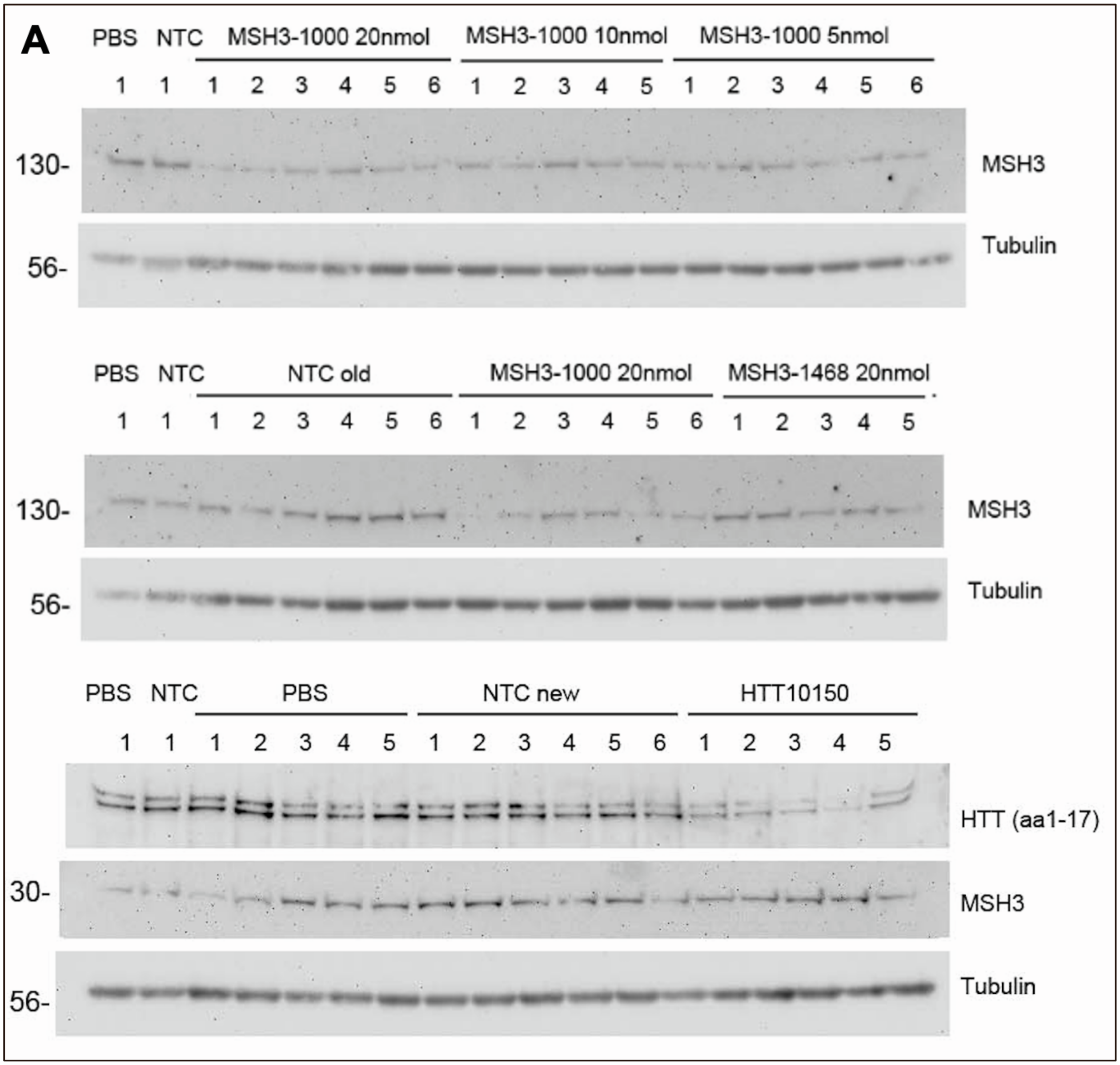

Figure S2: MSH3_1000 silences MSH3 *in vivo* two months post ICV injection in Hdh^{Q111} HD mice. Western blots used to quantify MSH3 and HTT protein expression in treated mice. HTT and MSH3 expression normalized to tublin loading control. Treatment group listed above each section. Molecular weight of target band reported on left. Western blot conditions reported in methods.

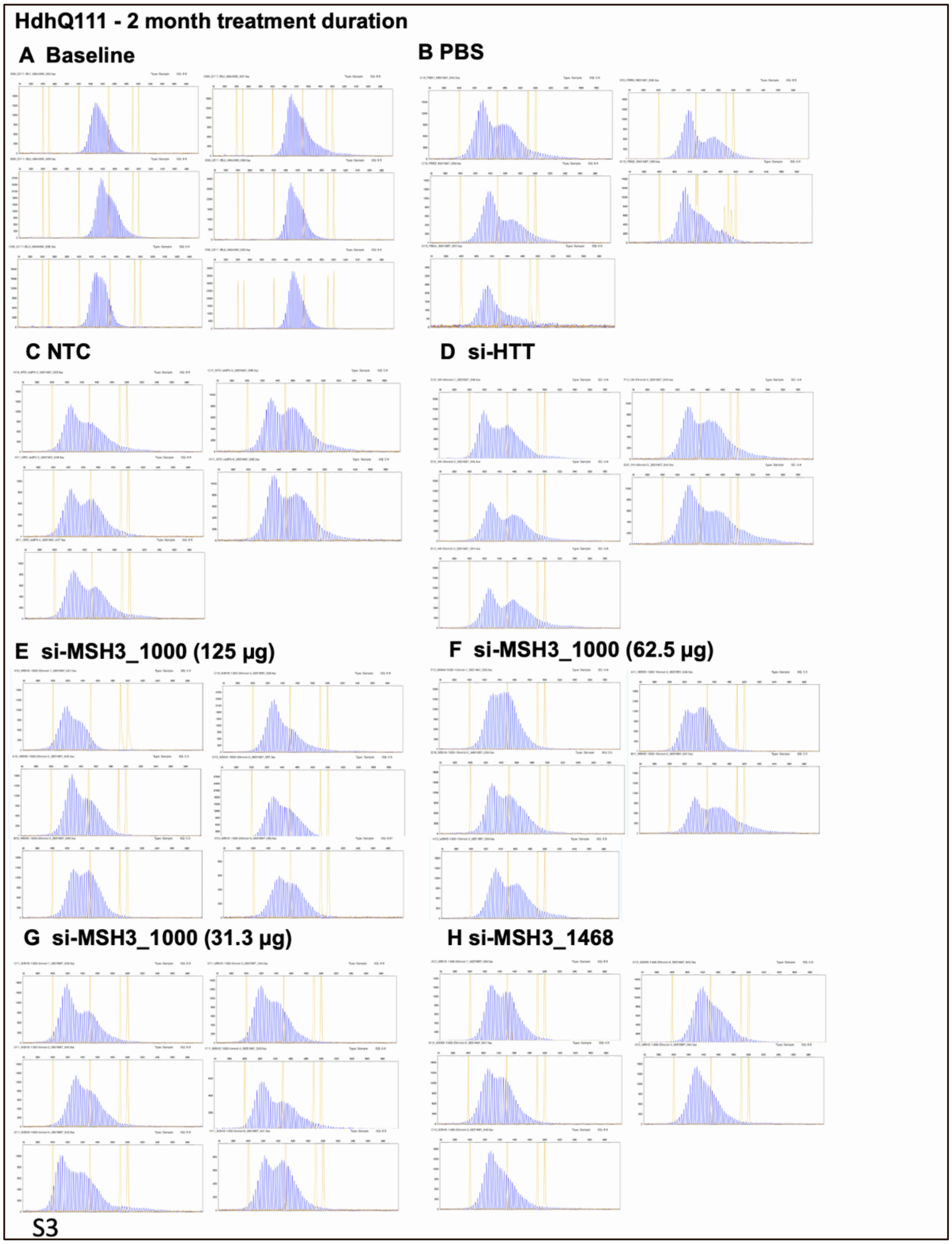

Figure S3: Fragment analysis traces indicate that siMSH3 blocks somatic repeat expansion in Hdh^{Q111} following two-month treatment. Fragment analysis traces used to quantify somatic repeat expansion instability index for (A) Baseline, (B) PBS, (C) NTC, (D) si-Htt (E) siMSH3_1000 (125 µg) (F) siMSH3_1000 (62.5 µg) (G) siMSH3_1000 (31.3 µg) (H) ssiMSH3_1468 (125 µg). X-asis of each chart is fragment length (base pairs). Y-axis is relative fluorescence units normalized to region of interest height. Orange lines are fragment ladders.

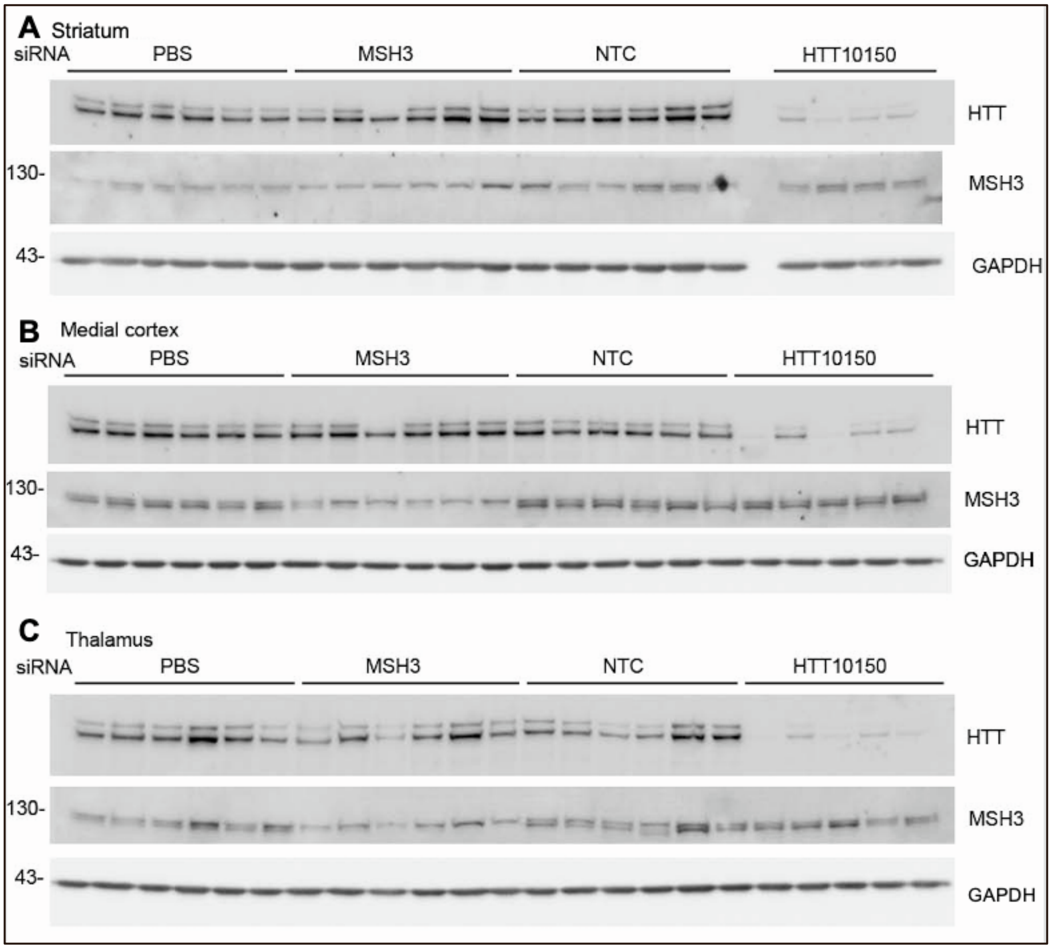

Figure S4: MSH3_1000 silences MSH3 *in vivo* two months post ICV injection in BAC-CAG HD mice. Western blots used to quantify MSH3 and HTT protein expression in **(A)** striatum, **(B)** medial cortex, **(C)** and thalamus. HTT and MSH3 expression normalized to GAPDH loading control. Treatment group listed above each section. Molecular weight of target band reported on left. Western blot conditions reported in methods.

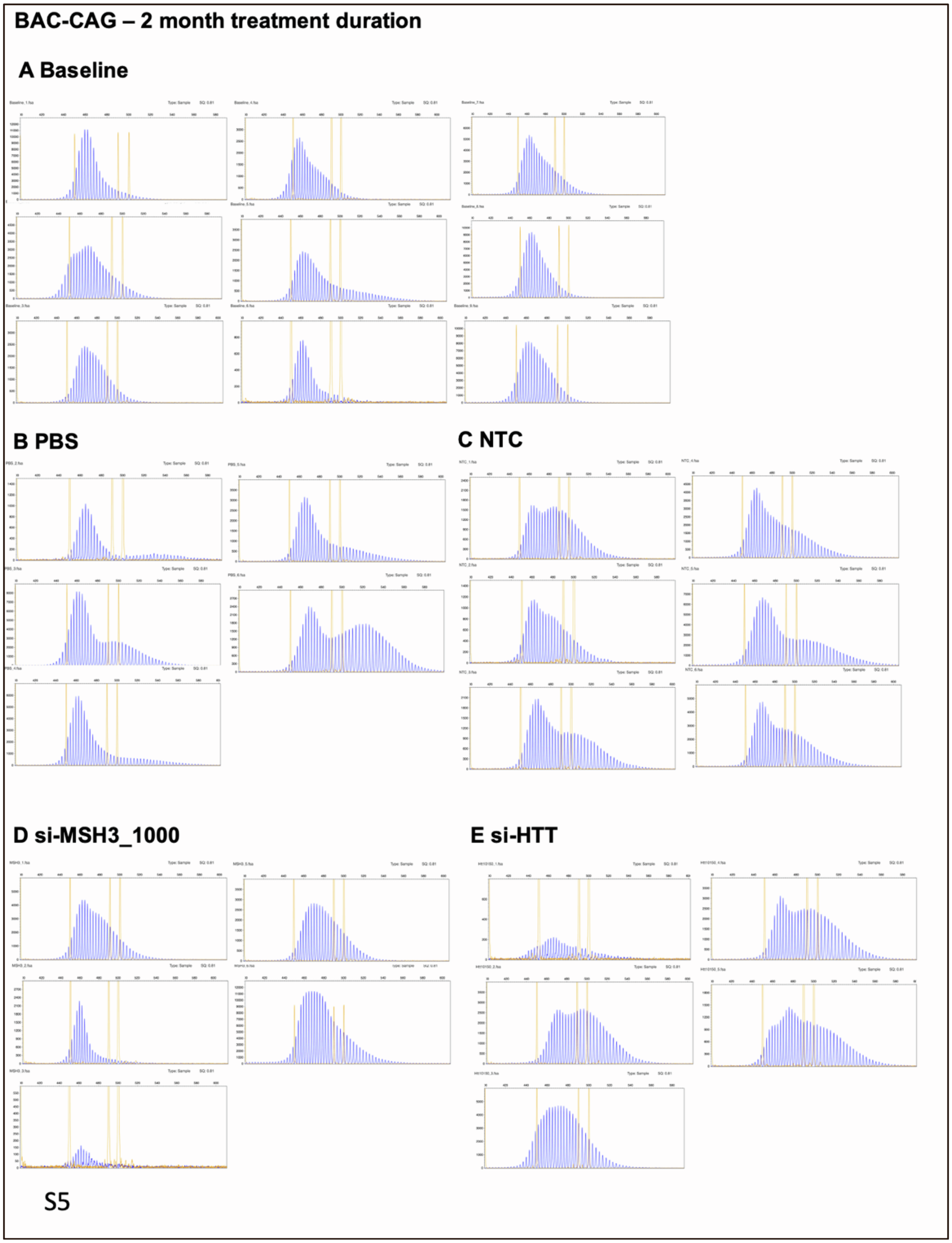

Figure S5: Fragment analysis traces indicate that siMSH3 blocks somatic repeat expansion in BAC-CAG following two-month treatment. Fragment analysis traces used to quantify somatic repeat expansion instability index for (A) Baseline, (B) PBS, (C) NTC, (D) siMSH3_1000 (E) si-Htt. X-axis of each chart is fragment length (base pairs). Y-axis is the relative fluorescence units normalized to region of interest height. Orange lines are fragment ladders.

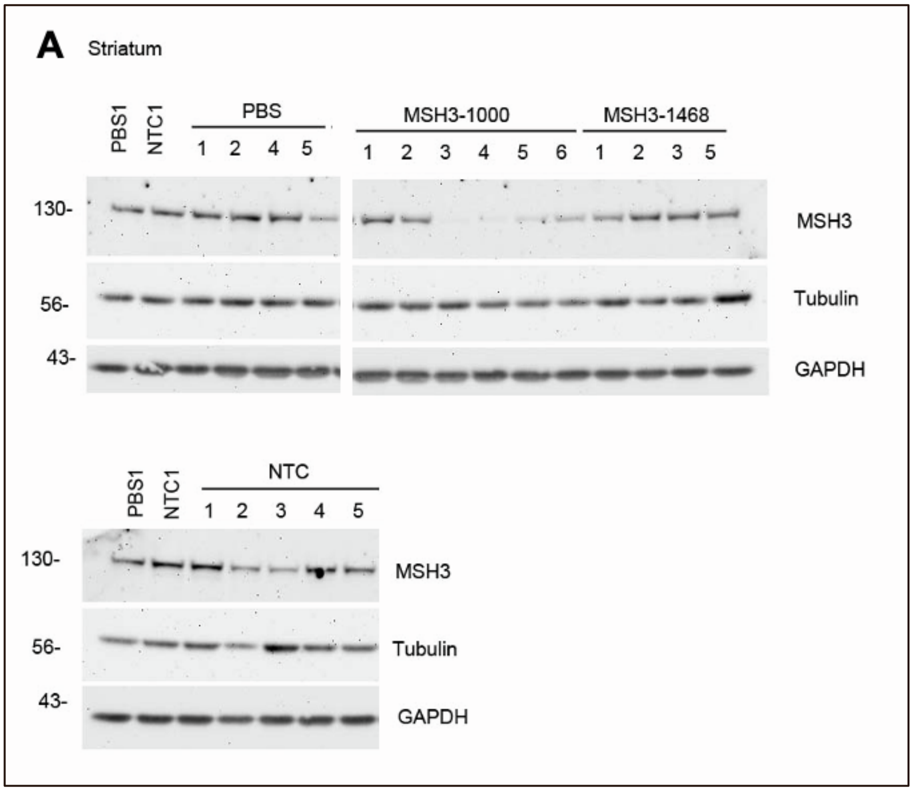

Figure S6: MSH3 targeting siRNA induce silencing *in vivo* four months post ICV injection in BAC-CAG HD mice. Western blots used to quantify MSH3 and HTT protein expression in **(A)** striatum. MSH3 expression normalized to GAPDH loading control. Tubulin used as an additional control. Treatment group listed above each section. Molecular weight of target band reported on left. Western blot conditions reported in methods.

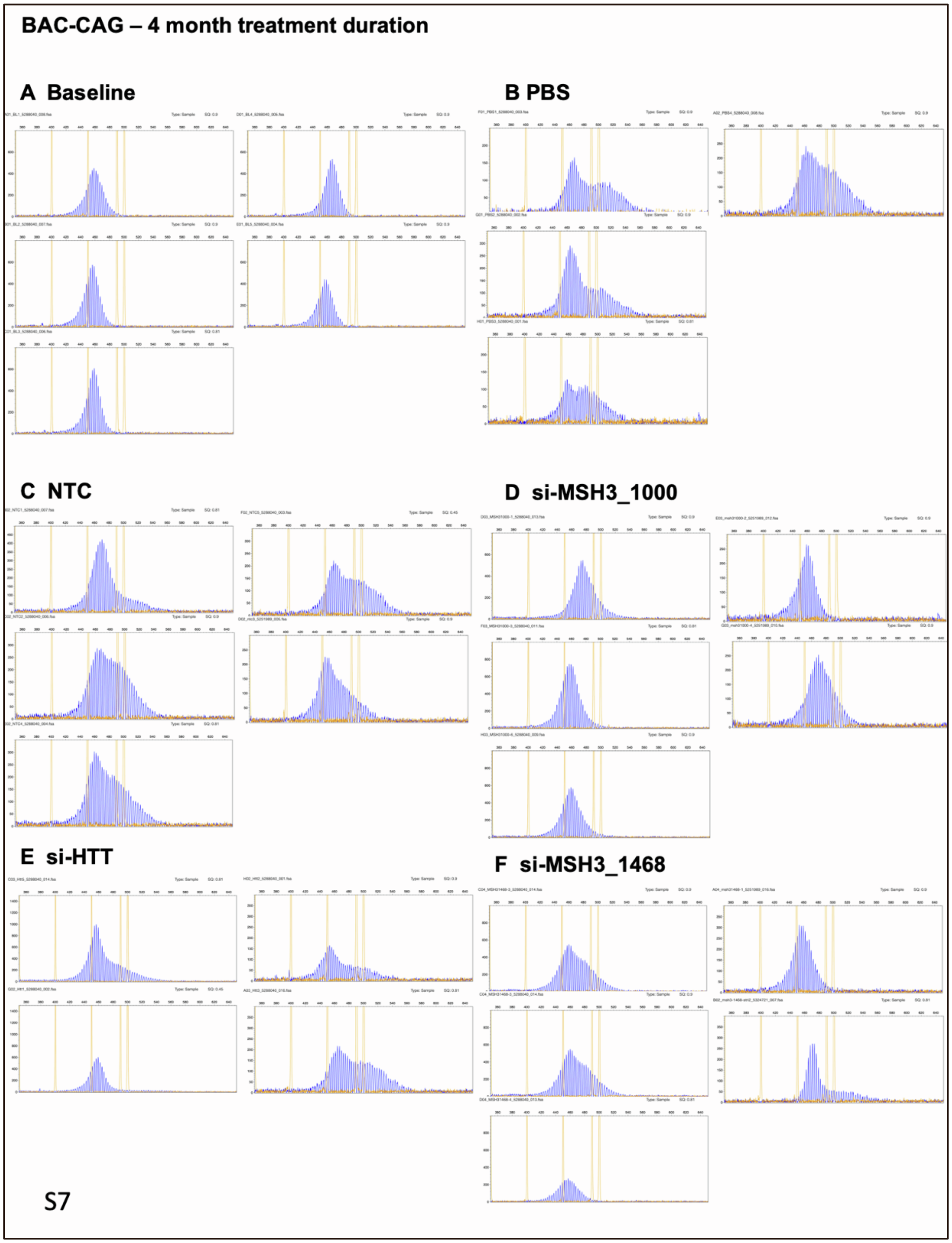

Figure S7: Fragment analysis traces indicate that siMSH3 blocks somatic repeat expansion in BAC-CAG following four-month treatment. Fragment analysis traces used to quantify somatic repeat expansion instability index for (A) Baseline, (B) PBS, (C) NTC, (D) siMSH3_1000, (E) si-Htt, (F) siMSH3_1468. X-axis of each chart is fragment length (base pairs). Y-axis is the relative fluorescence units normalized to region of interest height. Orange lines are fragment ladders.

Table S1: Summary of siRNA chemistries used for *in vitro* and *in vivo* studies

Compound	Guide strand chemistry (5'-3')	Passenger strand chemistry (5'-3')
MSH3_885	P(mU)#(fG)#(mG)(fC)(fA)(fA)(mU)(fA)(mA)(fA)(mU)(fA)(mU)#(fU)#(mG)#(fA)#(mG)#(mC)#(mU)#(fC)	(mC)#(mA)#(fA)(mU)(fA)(mU)(fU)(mU)(fA)(mU)(mU)(mG)(fC)#(mC)#(mA)-TegChol
MSH3_993	P(mU)#(fC)#(mA)(fG)(fU)(fU)(mU)(fG)(mC)(fU)(mU)(fC)(mA)#(fC)#(mA)#(fA)#(mC)#(mU)#(mC)#(fC)	(mU)#(mG)#(fU)(mG)(fA)(mA)(fG)(mC)(fA)(mA)(mA)(mC)(fU)#(mG)#(mA)-TegChol
MSH3_1000	P(mU)#(fG)#(mC)(fA)(fG)(fU)(mU)(fU)(mC)(fA)(mG)(fU)(mU)#(fU)#(mG)#(fC)#(mU)#(mU)#(mC)#(fA)	(mC)#(mA)#(fA)(mA)(fC)(mU)(fG)(mA)(fA)(mA)(mC)(mU)(fG)#(mC)#(mA)-TegChol
MSH3_1468	P(mU)#(fA)#(mA)(fA)(fG)(fC)(mA)(fU)(mG)(fG)(mC)(fU)(mG)#(fU)#(mA)#(fU)#(mU)#(mC)#(mA)#(fA)	(mU)#(mA)#(fC)(mA)(fG)(mC)(fC)(mA)(fU)(mG)(mC)(mU)(fU)#(mU)#(mA)-TegChol
MSH3_1763	P(mU)#(fA)#(mG)(fU)(fG)(fU)(mG)(fG)(mU)(fC)(mU)(fA)(mA)#(fA)#(mA)#(fC)#(mC)#(mC)#(mA)#(fC)	(mU)#(mU)#(fU)(mU)(fA)(mG)(fA)(mC)(fC)(mA)(mC)(mA)(fC)#(mU)#(mA)-TegChol
MSH3_1953	P(mU)#(fU)#(mG)(fU)(fG)(fA)(mU)(fA)(mA)(fA)(mU)(fG)(mC)#(fU)#(mA)#(fC)#(mA)#(mG)#(mA)#(fG)	(mU)#(mA)#(fG)(mC)(fA)(mU)(fU)(mU)(fA)(mU)(mC)(mA)(fC)#(mA)#(mA)-TegChol
MSH3_2048	P(mU)#(fG)#(mU)(fG)(fG)(fG)(mA)(fA)(mU)(fU)(mA)(fA)(mC)#(fA)#(mG)#(fC)#(mA)#(mG)#(mG)#(fU)	(mC)#(mU)#(fG)(mU)(fU)(mA)(fA)(mU)(fU)(mC)(mC)(mC)(fA)#(mC)#(mA)-TegChol
MSH3_2170	P(mU)#(fA)#(mA)(fU)(fA)(fA)(mU)(fU)(mC)(fA)(mG)(fU)(mU)#(fU)#(mU)#(fA)#(mU)#(mC)#(mC)#(fC)	(mA)#(mA)#(fA)(mA)(fC)(mU)(fG)(mA)(fA)(mU)(mU)(mA)(fU)#(mU)#(mA)-TegChol
MSH3_2204	P(mU)#(fC)#(mU)(fU)(fU)(fU)(mU)(fU)(mA)(fU)(mU)(fA)(mA)#(fA)#(mG)#(fG)#(mG)#(mA)#(mA)#(fG)	(mC)#(mU)#(fU)(mU)(fA)(mA)(fU)(mA)(fA)(mA)(mA)(mA)(fA)#(mG)#(mA)-TegChol
MSH3_2397	P(mU)#(fG)#(mG)(fC)(fU)(fC)(mA)(fC)(mA)(fG)(mC)(fU)(mU)#(fU)#(mU)#(fG)#(mU)#(mG)#(mC)#(fU)	(mA)#(mA)#(fA)(mA)(fG)(mC)(fU)(mG)(fU)(mG)(mA)(mG)(fC)#(mC)#(mA)-TegChol
MSH3_2675	P(mU)#(fC)#(mA)(fG)(fC)(fA)(mA)(fC)(mA)(fC)(mA)(fU)(mC)#(fA)#(mA)#(fU)#(mC)#(mA)#(mC)#(fA)	(mU)#(mU)#(fG)(mA)(fU)(mG)(fU)(mG)(fU)(mU)(mG)(mC)(fU)#(mG)#(mA)-TegChol
MSH3_2897	P(mU)#(fU)#(mA)(fU)(fA)(fU)(mU)(fG)(mU)(fC)(mU)(fG)(mC)#(fA)#(mG)#(fC)#(mA)#(mC)#(mC)#(fC)	(mC)#(mU)#(fG)(mC)(fA)(mG)(fA)(mC)(fA)(mA)(mU)(mA)(fU)#(mA)#(mA)-TegChol
MSH3_566	P(mU)#(fA)#(mU)(fC)(fA)(fG)(mU)(fA)(mC)(fA)(mU)(fU)(mU)(fU)#(mG)#(fG)#(mC)#(mA)#(mG)#(fA)#(mU)	(mC)#(mC)#(mA)(fA)(mA)(fA)(mU)(fG)(mU)(fA)(mC)(mU)(mG)(fA)#(mU)#(mA)-TegChol
MSH3_672	P(mU)#(fC)#(mA)(fA)(fA)(fC)(mU)(fG)(mA)(fC)(mU)(fG)(mA)(fG)#(mA)#(fU)#(mC)#(mA)#(mA)#(fA)#(mU)	(mA)#(mU)#(mC)(fU)(mC)(fA)(mG)(fU)(mC)(fA)(mG)(mU)(mU)(fU)#(mG)#(mA)-TegChol
MSH3_715	P(mU)#(fG)#(mA)(fA)(fG)(fC)(mA)(fG)(mU)(fU)(mU)(fU)(mC)(fU)#(mG)#(fU)#(mA)#(mA)#(mA)#(fU)#(mU)	(mA)#(mC)#(mA)(fG)(mA)(fA)(mA)(fA)(mC)(fU)(mG)(mC)(mU)(fU)#(mC)#(mA)-TegChol
MSH3_1452	P(mU)#(fC)#(mA)(fA)(fA)(fA)(mU)(fA)(mA)(fA)(mU)(fG)(mU)(fU)#(mA)#(fU)#(mC)#(mC)#(mA)#(fU)#(mU)	(mA)#(mU)#(mA)(fA)(mC)(fA)(mU)(fU)(mU)(fA)(mU)(mU)(mU)(fU)#(mG)#(mA)-TegChol
MSH3_1487	P(mU)#(fA)#(mA)(fA)(fC)(fU)(mC)(fU)(mG)(fU)(mA)(fA)(mC)(fU)#(mG)#(fC)#(mC)#(mU)#(mG)#(fG)#(mU)	(mG)#(mC)#(mA)(fG)(mU)(fU)(mA)(fC)(mA)(fG)(mA)(mG)(mU)(fU)#(mU)#(mA)-TegChol
MSH3_1505	P(mU)#(fA)#(mA)(fC)(fU)(fG)(mU)(fA)(mU)(fC)(mU)(fU)(mU)(fU)#(mG)#(fC)#(mA)#(mU)#(mA)#(fA)#(mU)	(mG)#(mC)#(mA)(fA)(mA)(fA)(mG)(fA)(mU)(fA)(mC)(mA)(mG)(fU)#(mU)#(mA)-TegChol
MSH3_1521	P(mU)#(fG)#(mA)(fG)(fA)(fA)(mC)(fC)(mU)(fU)(mU)(fG)(mA)(fU)#(mG)#(fU)#(mC)#(mA)#(mA)#(fC)#(mU)	(mA)#(mC)#(mA)(fU)(mC)(fA)(mA)(fA)(mG)(fG)(mU)(mU)(mC)(fU)#(mC)#(mA)-TegChol
MSH3_1548	P(mU)#(fC)#(mU)(fA)(fA)(fG)(mU)(fU)(mA)(fA)(mC)(fA)(mA)(fU)#(mG)#(fC)#(mC)#(mA)#(mG)#(fA)#(mU)	(mG)#(mC)#(mA)(fU)(mU)(fG)(mU)(fU)(mA)(fA)(mC)(mU)(mU)(fA)#(mG)#(mA)-TegChol
MSH3_1590	P(mU)#(fG)#(mG)(fU)(fA)(fU)(mU)(fU)(mU)(fA)(mU)(fG)(mA)(fU)#(mG)#(fG)#(mC)#(mA)#(mG)#(fC)#(mU)	(mC)#(mC)#(mA)(fU)(mC)(fA)(mU)(fA)(mA)(fA)(mA)(mU)(mA)(fC)#(mC)#(mA)-TegChol
MSH3_1647	P(mU)#(fA)#(mU)(fA)(fG)(fC)(mU)(fG)(mU)(fU)(mU)(fA)(mA)(fA)#(mA)#(fU)#(mU)#(mC)#(mU)#(fC)#(mU)	(mA)#(mU)#(mU)(fU)(mU)(fA)(mA)(fA)(mC)(fA)(mG)(mC)(mU)(fA)#(mU)#(mA)-TegChol
MSH3_1654	P(mU)#(fU)#(mU)(fA)(fC)(fU)(mU)(fG)(mA)(fU)(mA)(fG)(mC)(fU)#(mG)#(fU)#(mU)#(mU)#(mA)#(fA)#(mU)	(mA)#(mC)#(mA)(fG)(mC)(fU)(mA)(fU)(mC)(fA)(mA)(mG)(mU)(fA)#(mA)#(mA)-TegChol
MSH3_1665	P(mU)#(fU)#(mA)(fA)(fA)(fU)(mU)(fC)(mC)(fA)(mU)(fU)(mU)(fU)#(mA)#(fC)#(mU)#(mU)#(mG)#(fA)#(mU)	(mG)#(mU)#(mA)(fA)(mA)(fA)(mU)(fG)(mG)(fA)(mA)(mU)(mU)(fU)#(mA)#(mA)-TegChol
MSH3_1666	P(mU)#(fA)#(mU)(fA)(fA)(fA)(mU)(fU)(mC)(fC)(mA)(fU)(mU)(fU)#(mU)#(fA)#(mC)#(mU)#(mU)#(fG)#(mU)	(mU)#(mA)#(mA)(fA)(mA)(fU)(mG)(fG)(mA)(fA)(mU)(mU)(mU)(fA)#(mU)#(mA)-TegChol
MSH3_1675	P(mU)#(fU)#(mU)(fA)(fA)(fU)(mU)(fG)(mU)(fC)(mA)(fU)(mA)(fA)#(mA)#(fU)#(mU)#(mC)#(mC)#(fA)#(mU)	(mA)#(mU)#(mU)(fU)(mA)(fU)(mG)(fA)(mC)(fA)(mA)(mU)(mU)(fA)#(mA)#(mA)-TegChol
MSH3_1697	P(mU)#(fC)#(mA)(fG)(fA)(fU)(mU)(fC)(mC)(fU)(mU)(fA)(mA)(fU)#(mG)#(fU)#(mU)#(mG)#(mU)#(fU)#(mU)	(mA)#(mC)#(mA)(fU)(mU)(fA)(mA)(fG)(mG)(fA)(mA)(mU)(mC)(fU)#(mG)#(mA)-TegChol
MSH3_1903	P(mU)#(fU)#(mG)(fA)(fU)(fU)(mU)(fU)(mC)(fU)(mA)(fU)(mC)(fU)#(mG)#(fA)#(mC)#(mC)#(mA)#(fA)#(mU)	(mU)#(mC)#(mA)(fG)(mA)(fU)(mA)(fG)(mA)(fA)(mA)(mA)(mU)(fC)#(mA)#(mA)-TegChol
MSH3_1905	P(mU)#(fG)#(mA)(fU)(fG)(fA)(mU)(fU)(mU)(fU)(mC)(fU)(mA)(fU)#(mC)#(fU)#(mG)#(mA)#(mC)#(fC)#(mU)	(mA)#(mG)#(mA)(fU)(mA)(fG)(mA)(fA)(mA)(fA)(mU)(mC)(mA)(fU)#(mC)#(mA)-TegChol
MSH3_1980	P(mU)#(fA)#(mG)(fA)(fA)(fG)(mA)(fA)(mC)(fU)(mC)(fU)(mU)(fG)#(mG)#(fG)#(mU)#(mA)#(mG)#(fA)#(mU)	(mC)#(mC)#(mC)(fA)(mA)(fG)(mA)(fG)(mU)(fU)(mC)(mU)(mU)(fC)#(mU)#(mA)-TegChol
MSH3_2019	P(mU)#(fG)#(mA)(fA)(fA)(fU)(mU)(fC)(mU)(fG)(mA)(fC)(mU)(fU)#(mU)#(fA)#(mG)#(mG)#(mU)#(fG)#(mU)	(mU)#(mA)#(mA)(fA)(mG)(fU)(mC)(fA)(mG)(fA)(mA)(mU)(mU)(fU)#(mC)#(mA)-TegChol
MSH3_2036	P(mU)#(fA)#(mG)(fC)(fA)(fG)(mG)(fU)(mA)(fU)(mU)(fA)(mU)(fU)#(mG)#(fC)#(mU)#(mU)#(mG)#(fA)#(mU)	(mG)#(mC)#(mA)(fA)(mU)(fA)(mA)(fU)(mA)(fC)(mC)(mU)(mG)(fC)#(mU)#(mA)-TegChol

MSH3_2190	P(mU)#(fG)#(mG)(fA)(fA)(fG)(mU)(fC)(mA)(fG)(mA)(fA)(mA)(fG)#(mG)#(fU)#(mC)#(mU)#(mU)#(fU)#(mU)	(mA)#(mC)#(mC)(fU)(mU)(fU)(mC)(fU)(mG)(fA)(mC)(mU)(mU)(fC)#(mC)#(mA)-TegChol
MSH3_2199	P(mU)#(fU)#(mU)(fA)(fU)(fU)(mA)(fA)(mA)(fG)(mG)(fG)(mA)(fA)#(mG)#(fU)#(mC)#(mA)#(mG)#(fA)#(mU)	(mA)#(mC)#(mU)(fU)(mC)(fC)(mC)(fU)(mU)(fU)(mA)(mA)(mU)(fA)#(mA)#(mA)-TegChol
MSH3_2339	P(mU)#(fA)#(mG)(fA)(fG)(fU)(mU)(fC)(mU)(fU)(mU)(fA)(mU)(fU)#(mU)#(fC)#(mU)#(mA)#(mU)#(fC)#(mU)	(mG)#(mA)#(mA)(fA)(mU)(fA)(mA)(fA)(mG)(fA)(mA)(mC)(mU)(fC)#(mU)#(mA)-TegChol
MSH3_2713	P(mU)#(fA)#(mA)(fA)(fU)(fC)(mU)(fG)(mU)(fA)(mU)(fU)(mA)(fU)#(mU)#(fU)#(mG)#(mG)#(mG)#(fA)#(mU)	(mA)#(mA)#(mA)(fU)(mA)(fA)(mU)(fA)(mC)(fA)(mG)(mA)(mU)(fU)#(mU)#(mA)-TegChol
MSH3_2790	P(mU)#(fC)#(mA)(fA)(fC)(fU)(mU)(fG)(mU)(fU)(mU)(fU)(mA)(fU)#(mG)#(fU)#(mA)#(mG)#(mG)#(fA)#(mU)	(mA)#(mC)#(mA)(fU)(mA)(fA)(mA)(fA)(mC)(fA)(mA)(mG)(mU)(fU)#(mG)#(mA)-TegChol
MSH3_2975	P(mU)#(fC)#(mA)(fA)(fG)(fG)(mA)(fC)(mU)(fG)(mU)(fG)(mA)(fU)#(mG)#(fU)#(mU)#(mG)#(mC)#(fU)#(mU)	(mA)#(mC)#(mA)(fU)(mC)(fA)(mC)(fA)(mG)(fU)(mC)(mC)(mU)(fU)#(mG)#(mA)-TegChol
MSH3_3080	P(mU)#(fC)#(mA)(fG)(fG)(fG)(mU)(fU)(mA)(fA)(mG)(fG)(mA)(fU)#(mU)#(fU)#(mC)#(mA)#(mC)#(fA)#(mU)	(mA)#(mA)#(mA)(fU)(mC)(fC)(mU)(fU)(mA)(fA)(mC)(mC)(mC)(fU)#(mG)#(mA)-TegChol
MSH3_3261	P(mU)#(fU)#(mC)(fC)(fU)(fU)(mG)(fC)(mU)(fG)(mC)(fA)(mA)(fU)#(mU)#(fC)#(mC)#(mU)#(mC)#(fU)#(mU)	(mG)#(mA)#(mA)(fU)(mU)(fG)(mC)(fA)(mG)(fC)(mA)(mA)(mG)(fG)#(mA)#(mA)-TegChol
MSH3_3270	P(mU)#(fA)#(mU)(fC)(fC)(fA)(mU)(fA)(mA)(fC)(mU)(fC)(mC)(fU)#(mU)#(fG)#(mC)#(mU)#(mG)#(fC)#(mU)	(mC)#(mA)#(mA)(fG)(mG)(fA)(mG)(fU)(mU)(fA)(mU)(mG)(mG)(fA)#(mU)#(mA)-TegChol
MSH3_3401	P(mU)#(fC)#(mA)(fU)(fC)(fG)(mU)(fC)(mC)(fA)(mU)(fA)(mA)(fC)#(mU)#(fU)#(mU)#(mG)#(mC)#(fA)#(mU)	(mA)#(mA)#(mG)(fU)(mU)(fA)(mU)(fG)(mG)(fA)(mC)(mG)(mA)(fU)#(mG)#(mA)-TegChol
MSH3_3464	P(mU)#(fA)#(mG)(fA)(fA)(fG)(mU)(fC)(mU)(fG)(mU)(fG)(mU)(fU)#(mU)#(fC)#(mU)#(mU)#(mC)#(fC)#(mU)	(mG)#(mA)#(mA)(fA)(mC)(fA)(mC)(fA)(mG)(fA)(mC)(mU)(mU)(fC)#(mU)#(mA)-TegChol
MSH3_3559	P(mU)#(fA)#(mA)(fG)(fA)(fU)(mA)(fG)(mG)(fC)(mU)(fG)(mU)(fU)#(mA)#(fC)#(mU)#(mG)#(mG)#(fA)#(mU)	(mG)#(mU)#(mA)(fA)(mC)(fA)(mG)(fC)(mC)(fU)(mA)(mU)(mC)(fU)#(mU)#(mA)-TegChol
MSH3_3588	P(mU)#(fG)#(mU)(fC)(fA)(fU)(mA)(fA)(mU)(fU)(mU)(fU)(mA)(fU)#(mG)#(fC)#(mU)#(mC)#(mA)#(fC)#(mU)	(mG)#(mC)#(mA)(fU)(mA)(fA)(mA)(fA)(mU)(fU)(mA)(mU)(mG)(fA)#(mC)#(mA)-TegChol
MSH3_3621	P(mU)#(fA)#(mA)(fA)(fC)(fC)(mU)(fC)(mU)(fC)(mU)(fG)(mU)(fU)#(mU)#(fC)#(mC)#(mA)#(mA)#(fU)#(mU)	(mG)#(mA)#(mA)(fA)(mC)(fA)(mG)(fA)(mG)(fA)(mG)(mG)(mU)(fU)#(mU)#(mA)-TegChol
MSH3_3694	P(mU)#(fG)#(mG)(fA)(fA)(fG)(mU)(fC)(mU)(fA)(mU)(fU)(mC)(fA)#(mA)#(fG)#(mA)#(mG)#(mU)#(fG)#(mU)	(mC)#(mU)#(mU)(fG)(mA)(fA)(mU)(fA)(mG)(fA)(mC)(mU)(mU)(fC)#(mC)#(mA)-TegChol
MSH3_3715	P(mU)#(fA)#(mA)(fA)(fA)(fU)(mU)(fU)(mU)(fC)(mU)(fA)(mA)(fU)#(mU)#(fA)#(mC)#(mA)#(mA)#(fA)#(mU)	(mU)#(mA)#(mA)(fU)(mU)(fA)(mG)(fA)(mA)(fA)(mA)(mU)(mU)(fU)#(mU)#(mA)-TegChol
MSH3_3761	P(mU)#(fG)#(mG)(fA)(fA)(fU)(mU)(fA)(mU)(fA)(mU)(fU)(mC)(fU)#(mG)#(fC)#(mC)#(mA)#(mC)#(fU)#(mU)	(mG)#(mC)#(mA)(fG)(mA)(fA)(mU)(fA)(mU)(fA)(mA)(mU)(mU)(fC)#(mC)#(mA)-TegChol
MSH3_3818	P(mU)#(fU)#(mG)(fA)(fA)(fC)(mU)(fG)(mA)(fA)(mA)(fC)(mA)(fA)#(mA)#(fU)#(mA)#(mA)#(mA)#(fA)#(mU)	(mA)#(mU)#(mU)(fU)(mG)(fU)(mU)(fU)(mC)(fA)(mG)(mU)(mU)(fC)#(mA)#(mA)-TegChol
MSH3_3870	P(mU)#(fA)#(mU)(fU)(fU)(fU)(mA)(fG)(mU)(fU)(mC)(fA)(mA)(fU)#(mG)#(fG)#(mA)#(mU)#(mA)#(fG)#(mU)	(mC)#(mC)#(mA)(fU)(mU)(fG)(mA)(fA)(mC)(fU)(mA)(mA)(mA)(fA)#(mU)#(mA)-TegChol
MSH3_3876	P(mU)#(fA)#(mA)(fA)(fA)(fU)(mU)(fA)(mU)(fU)(mU)(fU)(mA)(fG)#(mU)#(fU)#(mC)#(mA)#(mA)#(fU)#(mU)	(mA)#(mA)#(mC)(fU)(mA)(fA)(mA)(fA)(mU)(fA)(mA)(mU)(mU)(fU)#(mU)#(mA)-TegChol
MSH3_4283	P(mU)#(fU)#(mU)(fG)(fA)(fU)(mA)(fA)(mU)(fU)(mC)(fU)(mA)(fU)#(mU)#(fU)#(mC)#(mU)#(mU)#(fU)#(mU)	(mA)#(mA)#(mA)(fU)(mA)(fG)(mA)(fA)(mU)(fU)(mA)(mU)(mC)(fA)#(mA)#(mA)-TegChol
MSH3_4315	P(mU)#(fC)#(mU)(fU)(fA)(fU)(mU)(fC)(mC)(fU)(mU)(fC)(mU)(fG)#(mU)#(fG)#(mC)#(mU)#(mC)#(fU)#(mU)	(mC)#(mA)#(mC)(fA)(mG)(fA)(mA)(fG)(mG)(fA)(mA)(mU)(mA)(fA)#(mG)#(mA)-TegChol
MSH3_4320	P(mU)#(fA)#(mU)(fG)(fA)(fC)(mC)(fU)(mU)(fA)(mU)(fU)(mC)(fC)#(mU)#(fU)#(mC)#(mU)#(mG)#(fU)#(mU)	(mA)#(mA)#(mG)(fG)(mA)(fA)(mU)(fA)(mA)(fG)(mG)(mU)(mC)(fA)#(mU)#(mA)-TegChol
MSH3_4336	P(mU)#(fU)#(mA)(fA)(fC)(fC)(mU)(fU)(mU)(fU)(mA)(fA)(mA)(fU)#(mU)#(fU)#(mC)#(mA)#(mU)#(fG)#(mU)	(mA)#(mA)#(mA)(fU)(mU)(fU)(mA)(fA)(mA)(fA)(mG)(mG)(mU)(fU)#(mA)#(mA)-TegChol
MSH3_4371	P(mU)#(fA)#(mC)(fA)(fA)(fU)(mC)(fU)(mU)(fU)(mA)(fA)(mA)(fC)#(mU)#(fG)#(mC)#(mU)#(mU)#(fA)#(mU)	(mC)#(mA)#(mG)(fU)(mU)(fU)(mA)(fA)(mA)(fG)(mA)(mU)(mU)(fG)#(mU)#(mA)-TegChol
MSH3_4372	P(mU)#(fA)#(mA)(fC)(fA)(fA)(mU)(fC)(mU)(fU)(mU)(fA)(mA)(fA)#(mC)#(fU)#(mG)#(mC)#(mU)#(fU)#(mU)	(mA)#(mG)#(mU)(fU)(mU)(fA)(mA)(fA)(mG)(fA)(mU)(mU)(mG)(fU)#(mU)#(mA)-TegChol
MSH3_4379	P(mU)#(fU)#(mU)(fC)(fA)(fU)(mC)(fC)(mA)(fA)(mC)(fA)(mA)(fU)#(mC)#(fU)#(mU)#(mU)#(mA)#(fA)#(mU)	(mA)#(mG)#(mA)(fU)(mU)(fG)(mU)(fU)(mG)(fG)(mA)(mU)(mG)(fA)#(mA)#(mA)-TegChol
MSH3_4410	P(mU)#(fA)#(mU)(fA)(fU)(fU)(mU)(fA)(mU)(fU)(mA)(fC)(mU)(fU)#(mG)#(fA)#(mA)#(mU)#(mG)#(fA)#(mU)	(mU)#(mC)#(mA)(fA)(mG)(fU)(mA)(fA)(mU)(fA)(mA)(mA)(mU)(fA)#(mU)#(mA)-TegChol
NTC_P3_*in vitro*	P(mU)#(fA)#(mA)(fU)(fC)(fG)(mU)(fA)(mU)(fU)(mU)(fG)(mU)#(fC)#(mA)#(fA)#(mU)#(mC)#(fA)#(mU)	(mG)#(mA)#(mU)(mU)(mG)(fA)(mC)(fA)(mA)(fA)(mU)(fA)(mC)(mG)(mA)(fU)#(mU)#(mA)-TegChol
MSH3_1000 _*in vivo*	V(mU)#(fG)#(mC)(fA)(fG)(fU)(mU)(fU)(mC)(fA)(mG)(fU)(mU)(fU)#(mG)#(fC)#(mU)#(mU)#(mC)#(fA)#(mU)	(mG)#(mC)#(mA)(fA)(mA)(fC)(mU)(fG)(mA)(fA)(mA)(mC)(mU)(fG)#(mC)#(mA)-DIO
HTT_10150_ *in vivo*	V(mU)#(fU)#(mA)(fA)(fU)(fC)(mU)(fC)(mU)(fU)(mU)(fA)(mC)(fU)#(mG)#(fA)#(mU)#(mA)#(mU)#(fA)#(mU)	(mU)#(mC)#(mA)(fG)(mU)(fA)(mA)(fA)(mG)(fA)(mG)(mA)(mU)(fU)#(mA)#(mA)-DIO
NTC_P3_AS _21mer	V(mU)#(fA)#(mA)(fU)(fC)(fG)(mU)(fA)(mU)(fU)(mU)(fG)(mU)(fC)#(mA)#(fA)#(mU)#(mC)#(mA)#(fU)#(mU)	(mU)#(mU)#(mG)(fA)(mC)(fA)(mA)(fA)(mU)(fA)(mC)(mG)(mA)(fU)#(mU)#(mA)-DIO
HTT_10150_ *in vivo 2*	V(mU)#(fU)#(mA)(fA)(fU)(fC)(mU)(fC)(mU)(fU)(mU)(fA)(mC)(fU)#(mG)#(fA)#(mU)#(mA)#(mU)#(mA)#(fU)	(mU)#(mC)#(mA)(fG)(fU)(fA)(mA)(fA)(mG)(fA)(mG)(mA)(mU)(fU)#(mA)#(mA)-DIO
NTC_P3_AS _21mer	V(mU)#(fA)#(mA)(fU)(fC)(fG)(mU)(fA)(mU)(fU)(mU)(fG)(mU)(fC)#(mA)#(fA)#(mU)#(mC)#(mA)#(mU)#(fU)	(mU)#(mU)#(mG)(fA)(mC)(fA)(mA)(fA)(mU)(fA)(mC)(mG)(mA)(fU)#(mU)#(mA)-DIO

Table S2: Summary of Mouse Strain, Genotype and CAG level
Please see the attached excel document for the full list of genotypes and mouse strain information
